# *Paraburkholderia tagetis* sp. nov., a novel species isolated from roots of *Tagetes patula* enhances the growth and yield of *Solanum lycopersicum* L. (tomato)

**DOI:** 10.3389/fmicb.2023.1140484

**Published:** 2023-04-04

**Authors:** Geeta Chhetri, Inhyup Kim, Jiyoun Kim, Yoonseop So, Sunho Park, Yonghee Jung, Taegun Seo

**Affiliations:** Department of Life Science, Dongguk University-Seoul, Goyang, Republic of Korea

**Keywords:** *Paraburkholderia*, polyhydroxybutyrate, plant growth promotion, phosphate solubilization, tomato (*Solanum lycopersicum* L.), *Tagetes patula*

## Abstract

A multifunctional, Gram-stain-negative, aerobic, motile by flagella, short-rod shaped bacteria, designated strain RG36^T^ was isolated from roots of marigold plant (*Tagetes patula*) sampled at Dongguk University, Republic of Korea. A 16S rRNA sequences indicated that the closest phylogenetic neighbors were *Paraburkholderia acidiphila* 7Q-K02^T^ (99.0%) and *Paraburkholderia sacchari* IPT101^T^ (98.9%) of the family Burkholderiaceae. The draft genome size was 8.52 Mb (63.7% GC). The genome contained 7,381 coding sequences. Digital DNA–DNA hybridization (dDDH) and average nucleotide identity (ANI) values of strain RG36^T^ with its most closely related species were only 83.1–88.7 and 27.6–36.7%, respectively. Strain RG36^T^ contained Q-8 as the major respiratory quinone and its main fatty acids (>10%) were C_16:0_, C_17:0_ cyclo, C_19:0_ cyclo ω8c, and summed feature 8 (comprising C_18:1_ ω7c and/or C_18:1_ ω6c). Strain RG36^T^ accumulates polyhydroxybutyrates (PHB) and exhibits multiple plant growth-promoting properties including production of indole-3-acetic acid (IAA), siderophores, protease, phosphate solubilization, and harboring gene clusters for its multifunctional properties. A pot experiment was conducted to evaluate the effect of PGPR on the growth of *Solanum lycopersicum* L. (Tomato). Result also confirmed the ability of strain RG36^T^ to promote tomato plant growth, especially it increases the yield of tomatoes. Structural assessment of the bioplastic by Fourier transform infrared (FTIR), nuclear magnetic resonance (NMR), and GC-MS spectroscopy, which confirmed the structure of the polymer as PHB. Our study revealed the potential of strain RG36^T^ to promote the growth of tomato plant and fruit yield by stimulating the various phytohormones, which could be use as bio-fertilizers to reduce the use of chemical fertilizers and promotes sustainable agricultural production. The phenotypic, chemotaxonomic and phylogenetic data, and genome analysis showed that strain RG36^T^ represents a novel species of the genus *Paraburkholderia*, for which the name *Paraburkholderia tagetis* sp. nov. is proposed. The type strain is RG36^T^ (=KACC 22685^T^ = TBRC 15696^T^).

## Introduction

Marigold (*Tagetes erecta* L.) is an economically important ornamental plant, well-known for its antimicrobial and medicinal properties (Gómez-Rodríguez et al., 2003). The plant is traditionally widely used for the treatment of skin disease, inflammatory swellings, wounds, oral disease, and urinary disorders (Verma et al., 2018). Horticulturists in temperate regions claim that the companion planting of marigolds with tomato (*Solanum lycopersicum* L.) plants protects tomatoes from glasshouse whitefly (Conboy et al., 2019). Marigold plants reduce pest populations directly by repellent volatile chemicals or indirectly by promoting beneficial arthropod populations in different types of plants (Jankowska et al., 2009; Sujayanand et al., 2016; Balzan, 2017; Zhao et al., 2017). Based on the evidence of the benefits of marigold plants to tomato plants when grown together, in this study we choose tomato plants to investigate the PGP ability of strain RG36^T^, which was originally isolated from the roots of marigold, near Dongguk University, Ilsan, Republic of Korea.

This is a continuation study from our previous work, where we isolated beneficial novel species from rhizosphere of marigold, among them *Chryseobacterium tagetis* RG1^T^ promotes rice plant growth and also showed antimicrobial activity against *Xanthomonas campestris* pv. *campestris* KACC 10377^T^ which is responsible for black rot disease of crucifers. Another species of *Lysobacter* produced exopolysaccharide (EPS), that inhibited biofilm formation, exhibited remarkably high antioxidant activity, and had excellent thermostability (Chhetri et al., 2022b; Kim et al., 2022).

For this study strain RG36^T^ was selected and I6S rRNA gene sequence similarity showed that the strain RG36^T^ belonged to the genus *Paraburkholderia*. The genus *Paraburkholderia*, was first proposed by Sawana with *Paraburkholderia graminis* as the type species, as the result of splitting the genus *Burkholderia* on the basis of phylogenomic evidence (Sawana et al., 2014). The majority of *Paraburkholderia* species have been isolated from plant roots or nodules and soil, although some have been found in aquatic environments and as opportunistic human pathogens (Herpell et al., 2021). Perhaps most notable is the ability of some to nodulate legumes and fix atmospheric nitrogen (Caballero-Mellado et al., 2004; Tapia-García et al., 2020). At the time of writing, the genus *Paraburkholderia* includes 128 species with validly published and correct names.^1^ Cells of the genus *Paraburkholderia* are Gram-stain-negative, straight, slightly curved, or sometimes coccoid rods, with one or more polar flagella (Brämer et al., 2001). Species of the genus *Paraburkholderia* usually carry large genomes (7–10 Mb) and are metabolically versatile. Studies on polyhydroxyalkanoate (PHA) production in the *Burkholderia* genus were initiated in the 1990s, and several *Burkholderia* species have been reported previously (Miotto-Vilanova et al., 2016). Among *Paraburkholderia* species, *Paraburkholderia xenovorans* LB400 and *Paraburkholderia sacchari* IPT101 produced polyhydroxybutyrate (PHB) using different carbon sources (Brämer et al., 2001; Urtuvia et al., 2018). The polyesters produced by strain RG36^T^ were characterized using FTIR, NMR spectroscopy, and GC-MS analysis and their functional groups were determined.

Previous studies showed that extensive root colonization by PHB producing strains and notably increased root area and the number of lateral roots compared to PHB-negative strains. The deletion of genes involved in PHB synthesis decreased the PGP ability of bacteria (Koller et al., 2005; Alves et al., 2019). PHB producing bacteria are able to resist stress conditions like carbon limitation, desiccation, heating, freezing-thawing, UV irradiation, and fluctuations in osmotic pressure (Kouřilová et al., 2021). 3-hydroxybutyrate acts as a chemical chaperon that can help the bacteria to survive under unfavorable conditions of oxidative stress and heat (Vinet and Zhedanov, 2011). PHB is produced in the form of water-insoluble granules within the cytoplasm of the cell and help microorganisms to survive under environmental stress conditions (Singh Saharan et al., 2014). During starvation, PHB protects the cellular components such as proteins and RNA (Vinet and Zhedanov, 2011). Similarly, it was recently reported that the presence of PHA granules, as well as a complex PHA metabolism, could enhance the robustness and resistance of bacterial cells to numerous stress factors, such as fluctuations in osmotic pressure, heating, freezing-thawing, UV irradiation, or oxidative pressure (Fuchs et al., 2011; Velázquez-Becerra et al., 2013; Aviles-Garcia et al., 2016; Martínez-Cámara et al., 2020; Ramírez-Ordorica et al., 2020). Based on these previous data, PHB produced by strain RG36^T^ could also help in PGP activity, stress resistance, carbon limitation, desiccation or other abiotic stressful conditions mentioned above.

Based on the evidence suggesting that the companion plant marigold plant protected many plants from pests, nematodes, and pathogenic microbes and promoted the growth of plants, we thought that beneficial bacteria in marigold roots must also be involved in the beneficial properties of marigold. Therefore, we identified and explored the multifunctional activities of *Paraburkholderia* sp. RG36^T^ isolated from marigold roots. In this study, we investigated the PHB synthesis and degradation and PGP activities of *Paraburkholderia* sp. RG36^T^ on tomato plants. Genome annotation of RG36^T^, revealed genes for bioplastic production, plant growth promotion, and synthesis of biosynthetic gene clusters. According to previous studies, PGP species should be good root colonizers, which make them good biocontrol agents against plant pathogens (Sandhya et al., 2009; Flemming, 2016). In our study, we also found that the selected isolate was able to produce biofilm under static conditions, which could help the strain in adhesion to roots for successful colonization, which is an essential characteristics to be a PBP bacteria. Here we, first report the species of *Paraburkholderia* that isolated from marigold rhizosphere. In addition this is the first novel species of this genus that has the tomato plant growth-promoting ability with high yield of tomatoes as compared to control.

## Materials and methods

### Isolation and ecology

There is not much information was available about the culturable rhizosphere microbial communities in the rhizosphere of marigold plants, therefore, in continuation of our previous work, for analyzing the culturable and beneficial bacterial communities in the roots of marigold plants, root samples were collected from the garden of Dongguk University, Goyang, Republic of Korea (37° 40′ 26.4″ N, 126° 48′ 20.88″ E). Roots samples were washed with tap water to remove the surface soil, then surface sterilized for 2 min in 70% ethanol, then 5 min in 3% sodium hypochlorite (NaOCl) solution, followed by three washes with sterile water, and then the surface moisture was absorbed with sterile filter paper. The roots were macerated with a sterile saline and shaken for 15 min on a shaker at 220 rpm, then used to generate a serial dilution up to 10^–5^. The spread plate technique was used to isolate the cultured bacteria. Strain RG36^T^ has been deposited in the Korean Agricultural Culture Collection (KACC 22685) and Thailand Bioresource Research Center (TBRC 15696).

### 16S rRNA phylogeny

The genomic DNA of strain RG36^T^ was extracted and PCR amplification and sequencing of 16S rRNA gene were performed as described previously (Kim et al., 2022). The 16S rRNA gene was amplified using universal primers 27F, 518F, 805R, and 1492R; and the PCR products were commercially sequenced (Solgent, Daejon, Republic of Korea). EzBioCloud’s Identify service was used to identify strain RG36^T^, and the 16S rRNA gene sequences of closely related type strains were retrieved. The draft genome sequence data have been deposited in the GenBank/EMBL/DDBJ and annotated using the NCBI Prokaryotic Genome Annotation Pipeline (Tatusov et al., 2000).

### Morphology and phenotypic characteristics

The morphology of cells grown on R2A agar at 30°C for 3 days was visualized under a transmission electron microscope (TEM) (LIBRA 120, Carl Zeiss, Germany). For this negative staining was done with 1% (w/v) phosphotungstic acid. The temperature range for growth was assessed using R2A medium by incubation at temperature ranging from 4–40°C (4, 10, 15, 20, 25, 28, 30, 35, 37, and 40°C). The tolerance of the cells to various NaCl concentrations was assessed in R2A broth containing 0–10% NaCl (w/v, at 0.5% intervals) at pH 7.0 for 10 days at 30°C. The growth of the cells on different media was assessed by incubation at 30°C for 5 days using R2A agar, marine agar (MA), nutrient agar (NA), trypticase soy agar (TSA), and Luria-Bertani agar (LB). Catalase and oxidase activities were tested using 3% (w/v) H_2_O_2_ and 1% (w/v) dimethylaniline, respectively. Gram staining was performed using the standard Gram stain method. Anaerobic growth was assessed by checking for colony formation on R2A agar after incubation at 30°C for 10 days in a GasPak jar (BBL, Cockeysville, MD, USA). Strain RG36^T^ was investigated for the production of four enzymes: protease, cellulase, lipase, and chitinase. For that R2A agar plate supplemented with the following macromolecules: casein (3% skimmed milk, w/v), carboxymethyl-cellulose (CM-cellulose; 1%, w/v), Tween 80 (1%, v/v), colloidal chitin (1%, w/v), and confirmed by the formation of distinct zones around the colonies after adding appropriate solutions or by direct observation as described previously (Kim et al., 2019; Kang et al., 2022). For other biochemical tests, API 20NE and API ZYM strips (bioMérieux) were used according to the manufacturer’s instructions.

### Chemotaxonomic characterization

For the analysis of cellular fatty acid methyl esters (FAME), cells of strain RG36^T^ and two reference strains were harvested from colonies grown on the third quadrants sectors of the plates after incubation on R2A for 3 days at 30°C. Cells were extracted by saponification, methylation and extraction using the MIDI protocol (Sherlock Microbial Identification System, version 6.0B). The FAME profile was analyzed with a gas chromatography (7890 GC; Agilent) using Sherlock Microbial Identification System version 6.1 (MIDI) with the TSBA6 database (Kuykendall et al., 1988). Respiratory quinone was extracted using chloroform/methanol (2:1, v/v), evaporated under a vacuum, re-extracted with acetone and analyzed using high-performance lipid chromatography (HPLC) in accordance with the protocol described in previous articles (Collins and Jones, 1981).

### *In vitro* screening of plant growth-promoting strain RG36^T^

Siderophore production was observed on Chrome-azurol S (CAS) plate assay as a yellow zone around the agar plug as described previously (Chhetri et al., 2021b). For qualitative assay of indole-3-acetic acid (IAA) production, strain RG36^T^ was inoculated in LB medium and incubated in a shaker (180 rpm, 25°C) for 3 days. 1 ml of the culture suspension was mixed with 2 ml of Salkowski reagent. Indication of pink color indicates IAA production. The intensity of the color was spectrophotometrically determined at 535 nm. Non-inoculated suspension was used as control. Phosphate solubilization was tested on a Pikovskaya’s medium (PKV) as described previously (Chhetri et al., 2021a). After 3 days, the halo zone around the colonies was observed. Strain RG36^T^ was also tested for nitrogen fixation in Jensen’s nitrogen-free agar media and incubated at 30°C for 10 days. The growth of colonies in Jensen’s agar plates indicated positive results for nitrogen fixation (Chhetri et al., 2021a). For biofilm production, RG36^T^ strain was inoculated from fresh R2A agar plates into LB broth and incubated for 48 h at 30°C under static conditions, as described in our recent work (Chhetri et al., 2022b).

### Tomato seed inoculation

To evaluate the effects of strain RG36^T^ inoculants on the growth of *S. lycopersicum* L. pot experiment was carried out in a greenhouse over 60 days. To this end, seeds of *S. lycopersicum* L. were treated with 70% ethanol for 1 min and 2% NaOCl containing 0.02% Tween 20 for 5 min in a 50 ml tube with vigorous shaking and washed three times with sterile distilled water (2 min each) (Subramanian et al., 2015). Then the seeds were germinated on sterile wet filter in Petri dishes at 25°C for 7 days in the dark.

After germination, seedlings of uniform size were soaked in bacterial suspensions for 2 h. The bacterial suspension was adjusted to OD_600 *nm*_ = 1, consistent with a density of nearly 10^7^ ∼ 10^8^ cells/ml. Seeds soaked in sterilized distilled water served as un-inoculated controls. Seven inoculated and un-inoculated seedlings (1:1) were transplanted into plastic pots containing sterilized soil and grown in the greenhouse of university. The planting soil was taken from nearby field of Dongguk University, Ilsan, Republic of Korea and used after disinfection under 121°C for 15 min. Each treatment had seven pots. 1 weeks later, inoculated seedlings was watered with 15 ml bacterial suspensions (10^8^ cells ml^–1^) again. The control pot was irrigated with water only, while the inoculated pot was irrigated with water every day and with 20 ml of RG36^T^ suspension (OD_600_ = 0.5) twice a week. After 60 days of growth, the plants were harvested and the roots were washed carefully with running water to remove the adherent soil and dried at room temperature for 4 h until no remaining water was visible on the surface. The lengths of the shoot and root and fresh and dry weights of the shoot and root were recorded with electronic scale and statistically analyzed using the *t*-test, respectively. The shoot and root parts were oven-dried at 60°C for 72 h.

### Staining for PHB detection

For the detection for PHB production by strain RG36^T^, lipophilic stain Sudan Black B. was used. Stain was prepared by dissolution of 0.3 gm powdered stain in 100 ml of 70% ethanol (Getachew and Woldesenbet, 2016). For microscopic observation, a thin bacterial smear was made and heat-fixed on clean glass slides, followed by staining with 0.3% solution of the Sudan Black B. After leaving the slides undisturbed for 15 min, counterstaining with safranin (5% w/v in sterile distilled water) was performed. Cells appearing blue-black under microscope were considered as PHB-positive strains.

### Extraction of PHB

Strain RG36^T^ was grown in 7 ml of R2A broth medium. After incubation for 24 h at 30°C, 2% (*v/v*) of the culture was aseptically transferred into 2 L conical flask containing 1 L of modified mineral salts medium (MSM) (pH 7.0) containing (in g/L) 20 glucose, 0.2 MgSO_4_, 0.1 NaCl, 0.5 KH_2_PO_4_, 4.0 peptone, and 2.5 yeast extract (Ramadas et al., 2009). Then, it was transferred to shaking incubator at 30°C (150 rpm). After 4 days, the culture broth was centrifuged at 5,000 rpm for 15 min. The supernatant was discarded and the pellet was dried. For the lysis of cells, NaOCl solution (12 ml) was added to the dried pellet and incubated for 2 h at 50°C. After incubation, it was centrifuged again at 5,000 rpm for 15 min and the supernatant was discarded. The pellet was washed with distilled water, acetone and methanol. The final polymeric particles were liquefied with addition of 5 ml of boiling chloroform and sieved by Whatman No. 1 filter paper. The chloroform was evaporated and PHB film was dried for 2 days in room temperature. The dried PHB film was stored for further analysis (Ramadas et al., 2009).

### FTIR spectrophotometer analysis

The instrument used for this analysis was a Nicolet iS50 FTIR spectrophotometer (Thermo Fisher Scientific). The PHB film was kept in the sample holder, and the spectra were recorded in the range of 4,000–400 cm^–1^. Obtained results were analyzed for the determination of functional groups.

### ^1^H NMR and ^13^C NMR analysis

For NMR analysis, around 3 mg of the purified PHB was dissolved in 1 ml of deuterated chloroform (CDCl_3_) at a concentration of 25 mg/ml using tetramethylsilane as an internal chemical shift reference. The ^1^H-NMR spectra were recorded at 500 MHz on a Bruker AVANCE 500 (NC, USA) spectrometer at 30°C. All NMR measurements were acquired at 298 K (25°C). Data were analyzed using Topspin 3.5 pl 7 software (Bruker BioSpin, GmbH Rheinstetten, Germany). Chemical shifts (δ) are expressed in ppm with reference to the residual solvent signals. Scalar coupling constants (J) are given in Hertz. The following conditions were used to record the ^1^H NMR and ^13^C-NMR spectra: 30°C pulse experiment; 3.28 s acquisition time; 1.10 s relaxation delay; 15.1 ppm (8,012 Hz) sweep width; 65,536 data points; and 2 dummy scans. The data were processed using line broadening 0.30 Hz.

### GC-MS analysis

GC-MS analysis of the sample was carried out after methanolysis of PHB (Danial et al., 2021). In brief, for methanolysis, the polymer sample was suspended in 1 ml chloroform and then 1 ml methanol containing 2.8 M H_2_SO_4_ in a screw-capped tube, and incubated at 100°C for 2 h. After cooling, 0.5 ml of demineralized water was added, and then the organic phase containing the resulting methyl esters of 4-hydroxyalkanoic acids was analyzed using GC-MS-ISQ LT (USA). The column used was a VF-5MS, 30 m × 0.250 mm dia with the film thickness of 0.25 μm and the column oven was programmed between 70 and 300°C at the rate of 10°C/min with the injection temperature of 280°C. Mass spectra were recorded in scan mode in the range of 40–1,000 m/z. Compounds were identified using NIST11. L library.

### Photodegradation of PHB in UV-light exposure and in soil

Biodegradation of PHB films exposed separately to UV light at 254 nm (UV-C band) in clean bench HB-402 and soil microbes. The corresponding control films (untreated with UV light and soil incubation) were evaluated. For both experiments, a piece of PHB film placed at room temperature served as the control. Photodegradation was followed by assessing the morphological changes after 2 weeks of exposure to UV light continuously. For the degradation experiment in soil, soil was collected from nearby field and filled into a Petri dish. A piece of PHB film was placed at the center of the soil and covered with soil. After rapping the Petri dish, it was incubated at 30°C. Results were recorded daily for 6 days. All PHB films were coated with platinum using ion sputtering equipment (15 nm; EM ACE200, Leica) and observed under a scanning electron microscope (FESEM; SIGMA).

### Genome sequencing and annotations

Whole-genome-based approaches were used for further analysis of *Paraburkholderia* sp. RG36^T^ strain. Genomic DNA of strain was extracted using the TaKaRa MiniBEST Bacteria Genomic DNA extraction Kit version 3.0 (TaKaRa) following the manufacturer’s instructions. The quality and quantity of the extracted genomic DNA (gDNA) were tested using the NanoDrop Spectrophotometer. A genomic DNA library was prepared using the TruSeq Nano DNA Prep Kit (Illumina). The draft genome was sequenced on the Illumina platform while the genome was assembled using the SPAdes genome assembler version 3.13.0 (Macrogen, Seoul, Republic of Korea). Functional annotation was conducted using the Evolutionary Genealogy of Genes: Non-supervised Orthologous Groups (eggNOG) 4.5 database (Huerta-Cepas et al., 2017). Genomic circular feature map was constructed using CGView server (Grant and Stothard, 2008). The CheckM bioinformatics tool was used to assess genome contamination and completeness^2^ of strain RG36^T^ (Parks et al., 2015). The genomic DNA G + C content was determined directly from the draft genome sequence. A phylogenomic tree was reconstructed on the basis of the concatenation of 92 core genes obtained from the close relatives of strain RG36^T^ in the genus *Paraburkholderia* (Na et al., 2018). To evaluate the intergenomic distances between genome sequences of strain RG36^T^ and its close strains belonging to the phylogenetically closest *Paraburkholderia* species, the FastANI and digital DNA–DNA hybridization (dDDH) values were determined (Meier-Kolthoff et al., 2013; Jain et al., 2018). The cut-off values of 96 and 70% were assigned as a species-delineation framework for ANI and digital DDH, respectively (Goris et al., 2007). Comparisons of orthologous gene clusters among strain RG36^T^ and other close strains were performed by using OrthoVenn2 (Xu et al., 2019). Biosynthetic gene clusters (BGCs) in bacterial genome sequences were identified and analyzed using antiSMASH v5.0.0 software (Blin et al., 2019). The genomic sequence was submitted to the NCBI GenBank Genome database under the accession number JAKLJA000000000.

## Results

### Phylogenetic analysis

**FIGURE 1:**
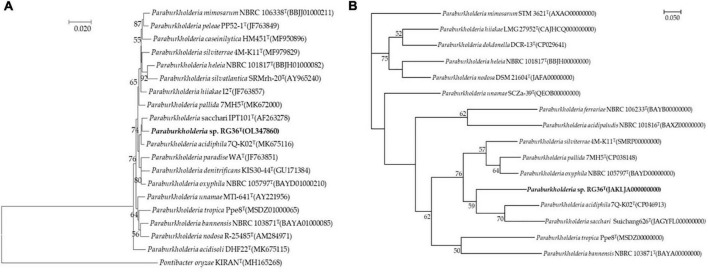
Neighbor-joining tree based on 16S rRNA gene sequences showing the relationship between strain RG36^T^ and related species. *Pontibacter oryzae* KIRAN^T^ (MH165268) was used as an out-group. Bootstrap values (based on 1,000 replications) greater than 50% are shown at branch points. Bar, 0.020 substitutions per nucleotide position **(A)**. Phylogenomic tree of strain RG36^T^ and closely related strains based on core genomes was constructed using UBCG, all genomes of 15 related strains were available on NCBI GenBank. GenBank accession numbers are shown in parentheses. Bootstrap analysis was carried out using 100 replications. Percentage bootstrap values (>50%) are given at branching points. Bar, 0.050 substitution per position **(B)**.

Comparative sequence analyses using the almost-complete 16S rRNA gene sequence (1,462 bp) of strain RG36^T^ revealed that strain was affiliated within the genus *Paraburkholderia*. According to 16S rRNA gene sequence similarities, the strain RG36^T^ was most closely related to *P. acidiphila* 7Q-K02^T^ (99.0%, 16S rRNA gene sequence similarity), followed by *P. sacchari* IPT101^T^ (98.9%), *Paraburkholderia guartelaensis* CNPSo 3008^T^ (98.6%), *Paraburkholderia hiiakae* 12^T^ (98.5%), and *Paraburkholderia paradise* WA^T^ (98.3%). The phylogenetic tree reconstructed using the neighbor-joining (NJ) algorithm (Figure 1A), which was subsequently compared with the maximum-parsimony (not shown) and maximum-likelihood trees (not shown), indicated that strain RG36^T^ affiliated within the genus *Paraburkholderia* and forms distinct clade with its most close relatives *P. acidiphila* 7Q-K02^T^ and *P. sacchari* IPT101^T^. These results suggested that strain RG36^T^ represent a novel species of the genus *Paraburkholderia*. Based on 16S rRNA gene sequence and phylogenetic analysis *P. acidiphila* 62472^T^ was purchased from Korean Collection for Type Culture (KCTC) and *P. sacchari* IPT101^T^ was a kind gift from Jiyoun Kim, Korea Research Institute of Bioscience and Biotechnology (KRIBB), Republic of Korea. For physiology, biochemical, fatty acid analyses, and quinone, the reference strains were selected and analyzed under identical conditions except where otherwise stated.

### Phenotypic, physiological, and chemotaxonomic characterization

Cells of strain RG36^T^ were Gram-stain negative, aerobic, motile by one polar flagellum, and rod-shaped, with dimensions of 0.6–1.0 μm in width and 1.3–2.5 μm in length (Supplementary Figure 1A). Colonies of strain RG36^T^ were white, thick, convex, and smooth with entire margins. Strain RG36^T^ was able to grow at 4–35°C (optimum, 28–30°C), pH 5.5–9.0 (pH 6.5–7.5) and in the presence of 0–3% (w/v) NaCl (0%, w/v). Strain RG36^T^ was positive for both catalase and oxidase activities. Grew well in R2A, NA, and TSA, however, no growth was occurred in MA and LB agar plates. Appearance of clear zone around the colony confirmed that strain RG36^T^ was able to hydrolyze casein (protease) and absence of clear zone confirmed the strain did not hydrolyze CM cellulose, Tween 80 and chitin. Previous studies showed that protease enzyme activity can boost the immune system of plant and allow them to withstand in stress full conditions (Figaj et al., 2019). Production of proteases by strain RG36^T^ could make it a promising disease suppressor and plant growth enhancer when they colonize the roots or vicinities of plant roots. The differential phenotypic characteristic features of strain RG36^T^ are presented in Table 1 with its two reference strains used in this study. Having the different colony color, oxidase reaction ability, hydrolysis of esculin, gelatin and casein, glucose fermentation, indole production, arginine dihydrolase ability, assimilation of D-mannitol and positive activities of α-chymotrypsin, trypsin, and acid phosphatase clearly differentiate the strain RG36^T^ from its close strains (Table 1).

**TABLE 1:** Differential characteristics of strain RG36^T^ in comparison with closely related species of *Paraburkholderia* species.

Characteristics	1	2	3
Isolation source	Rhizosphere	Soil	Soil
Colony color	White	Light yellow	Light beige
Catalase/oxidase	**+/+**	**±**	**±**
Temperature range for growth (°C)	4–35	4–37	5–40
NaCl range for growth (%)	0–3	0–3.5	0–3
pH range for growth	5.5–9.0	3.0–8.5	4.0–8.0
**Hydro:**			
Esculin	+	+	–
Gelatin	+	–	–
Casein	+	+	–
Glucose fermentation	+	–	–
Indole production	+	–	–
Arginine dihydrolase	+	–	–
**Assimilation (API 20NE) of:**			
D-glucose	–	–	+
D-mannitol	+	+	–
*N*-acetyl-D-glucosamine	–	+	+
Capric acid	–	–	+
Trisodium citrate	–	+	+
**Enzyme activities (API ZYM)**			
α-Chymotrypsin	+	–	–
Cystine arylamidase	–	+	–
Trypsin	+	–	–
Valine arylamidase	–	+	+
Acid phosphatase	+	+	–
β-Galactosidase	–	+	+
β-Glucosidase	–	+	+
DNA G + C content (mol%)	63.7	64.3	64

Strain: 1, RG36^T^; 2, *P. acidiphila* 7Q-K02T; 3, *P. sacchari* IPT101^T^. All data are from this study except GC content for two reference strain. GC content of *P. acidiphila* 7Q-K02^T^ and *P. sacchari* IPT101^T^ are from NCBI.

The sole respiratory quinone was Q-8, the same as the major respiratory quinone reported for the other members of the genus *Paraburkholderia*. The major cellular fatty acids (>10%) of strain RG36^T^ were C_16:0_, C_17:0_ cyclo, C_19:0_ cyclo ω8c, summed feature 8 (comprising C_18:1_ ω7c and/or C_18:1_ ω6c). The major fatty acids of the other close strains of the genus *Paraburkholderia* are similar, however, the difference between the composition of major fatty acids between the strain RG36^T^ and its reference strains distinguish it from other species of genus *Paraburkholderia*. In addition the presence and absence of C_16:1_ 2-OH and iso C_17:0_ 3-OH differentiate it from its close two reference strains (Supplementary Table 1). Therefore, the chemotaxonomic profile of the novel strain is in accordance with other *Paraburkholderia* species, corroborating their inclusion into the genus *Paraburkholderia*.

### Plant growth promoting traits

Strain RG36^T^ was found to be positive for production of IAA, siderophore production, phosphate solubilization, and nitrogen fixation. The observed color change showed that this strain had the ability to synthesize IAA only in the presence of the precursor L-tryptophan and could produce 12.5 μg/ml IAA. Strain RG36^T^ showed a halo surrounding the individual colonies, indicating siderophore production and iron quenching from the dye complex. This result confirmed that the strain RG36^T^ is able to dissolve iron and produce siderophore. For nitrogen fixation, the *Escherichia coli*, which is unable to grow on nitrogen-free medium, was used as a negative control. Only strain RG36^T^ showed a good growth in nitrogen medium after 4 days of incubation at 30°C, which confirmed the nitrogen fixation ability of this strain. Previous studies also confirmed that the species of *Paraburkholderia* are able to nodulate legumes and fix atmospheric nitrogen (Tapia-García et al., 2020). Strain RG36^T^ produced a clear zone around the colony in PVK agar plates. This was a clear indication of its ability to utilize inorganic phosphate in the medium (Supplementary Figure 1B). Cells formed biofilms, which was confirmed by the presence of floating pellicles in the liquid media. The ability to form biofilms enhances bacterial survival, colonization, and plant growth through the various mechanisms (Sandhya et al., 2009; Flemming, 2016).

### Effect of *Paraburkholderia* strain RG36^T^ on the growth of tomato plant

**FIGURE 2:**
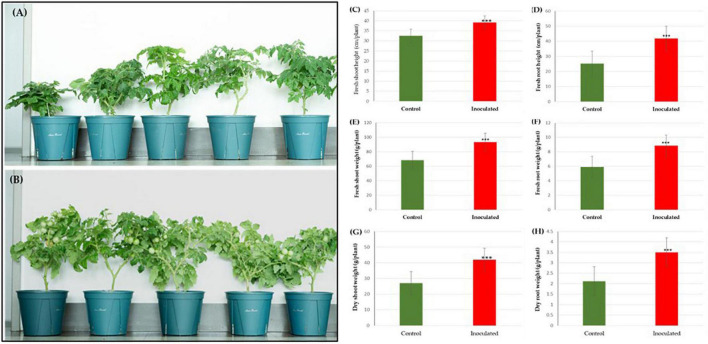
Effects of *Paraburkholderia* sp. RG36^T^ inoculation on the growth of tomato seedlings after 60 days of incubation. Representative images of seedlings growing on control **(A)** and inoculated pots in green house **(B)**. On average, fresh shoot height **(C)**, fresh root height **(D)**, fresh shoot weight **(E)**, fresh root weight **(F)**, dry shoot weight **(G)**, and dry root weight **(H)** increased by 20, 79.2, 36.6, 139, 64.2, and 73.1%, respectively. Fresh plant biomass **(C)** increased by 89.6% and dry plant biomass **(D)** by 97.1% (average from 5 explants ± SD, ***p ≤ 0.01).

**FIGURE 3:**
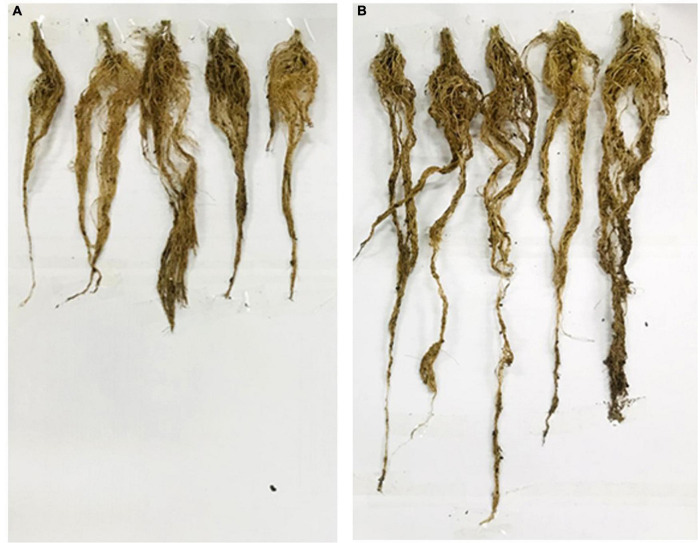
Phenotypic expression of the tomato plant roots: Non-inoculated control plants **(A)**. Root length of RG36^T^ treated plants was also significantly higher than non-inoculated control plants **(B)**.

**FIGURE 4:**
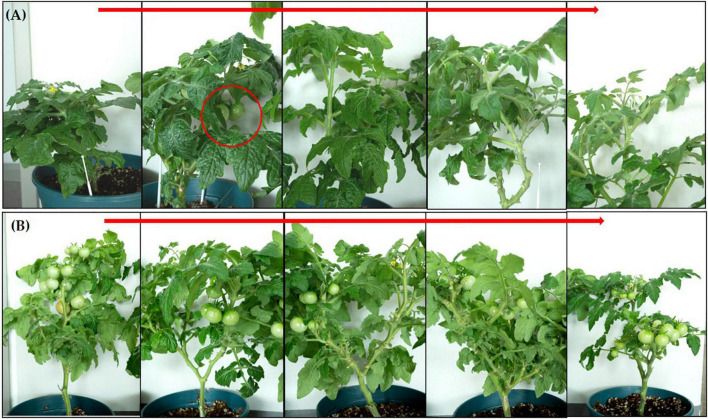
Increase in fruit yield found in tomato plants when inoculated with *Paraburkholderia* species RG36^T^. **(A)** Control or non-treated plants showed only three fruits among five plants. However, inoculated plants showed more than ten tomatoes yield in each plant **(B)**.

In the pot experiment, our study revealed that a significant improvement in shoot length, shoot fresh and dry weights, root length and root fresh, and dry weights of tomato plants due to inoculation of strain RG36^T^ as compared to control or untreated seedlings which is shown in Figure 2A (control) and Figure 2B (treated). The data obtained indicated that strain RG36^T^ possessed functional plant-growth-promoting traits that positively impacted tomato growth and development. When compared to the control groups, the seeds inoculated with the RG36^T^ increased in fresh shoot length, fresh root length, fresh shoot weight, fresh root weight, dry shoot weight, and dry root weight by 20, 79.2, 36.6, 139, 64.2, and 73.1%, respectively. The different growth parameters of the control and inoculated tomato plants are illustrated in Figures 2C–H. There was a huge difference in the length of dry roots that had been treated with strain RG36^T^ in all five pots compared to the un-treated plant (Figures 3A, B). Most notably, we found that inoculation of strain RG36^T^ in tomato plants increased fruit yield compared to the control. Among five plants, the controls yielded just three tomatoes, whereas plants treated with strain RG36^T^ yield more than ten tomatoes per plant (Figures 4A, B). Crop yield was evaluated by measuring fruit number. Inoculation of *Burkholderia* species MTo-293 has been shown to increases fruit yield in tomato plants upon significant colonization of strain to aerial tissues in previous study (Vio et al., 2022). Hence, in our study, the increase in fruits yield only in treated plants suggests that strain RG36^T^ moved to the aerial tissues and its colonization was accompanied by an enhancement of tomato production in all five treated plants compared to their control groups.

The enhancement of root growth can be associated with auxin and siderophore production, nitrogen-fixation, and phosphate solubilization by strain RG36^T^. Auxins are known to stimulate the growth of lateral roots and our results were consistent with the previous reports on the PGP abilities of several species of the *Paraburkholderia* genus (Issa et al., 2018; Gallart et al., 2022; Vio et al., 2022).

Siderophore produced by *Pseudomonas fluorescens* enriched the ferric iron as nutrients and that enhanced photosynthetic pigments and biomass of the tomato plant (Singh et al., 2022). So siderophore produced by novel strain also played a vital role in plant growth enhancement. Moreover, the fixation of more nitrogen in roots by strain RG36^T^ could also allowed improved growth and development in roots length and weight.

Phosphate solubilizing bacteria (PSB) constitute an important microbial taxon that converts insoluble phosphorus into soluble forms which is then absorb by plants. PSB lower the pH of the micro zone in the roots and release phosphorus from insoluble rock phosphate (inorganic form) and insoluble organophosphorus (organic form) by secreting organic acids, acid phosphatase, and alkaline phosphatase and improves the absorption of phosphorus by plants (Calvo et al., 2010; Chauhan et al., 2015; Urtuvia et al., 2018; Batool and Iqbal, 2019). The member of the genus *Paraburkholderia* usually has a strong capacity to solubilize inorganic phosphates. Based on these reports, our study suggest that phosphate solubilization property of strain RG36^T^ also played its vital role is growth enhancement of tomato plant.

### Staining and extraction of PHB

Sudan black is the specific stain to color various lipids, such as phospholipids, neutral lipids, and sterols, therefore it was used to screen for PHB producing bacteria. Observation of the Sudan Black B staining result under a light microscope showed the presence of granules (black section) which confirmed the production of PHB by strain RG36^T^ when growth is nutrient limited (Supplementary Figure 1C). After 48 h of incubation in the presence of glucose as a carbon source at 30°C, crude extract of PHB was dried at room temperature. Next day the crude extract was turns into white plastic film, which was later quantified by FTIR, NMR, and GC-MS analysis. The weight of plastic film was found to be 0.65 g/L when measured in electronic balance, which is very less amount. Therefore, the optimization of media for production of PHB in a good amount in required, and we are working on it.

### FTIR analysis

**FIGURE 5:**
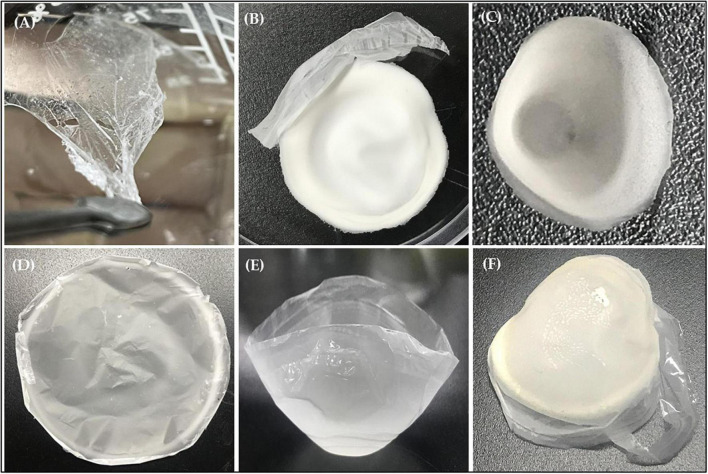
Appearance of biodegradable plastic produced from glucose **(A–F)** by strain RG36^T^.

**FIGURE 6:**
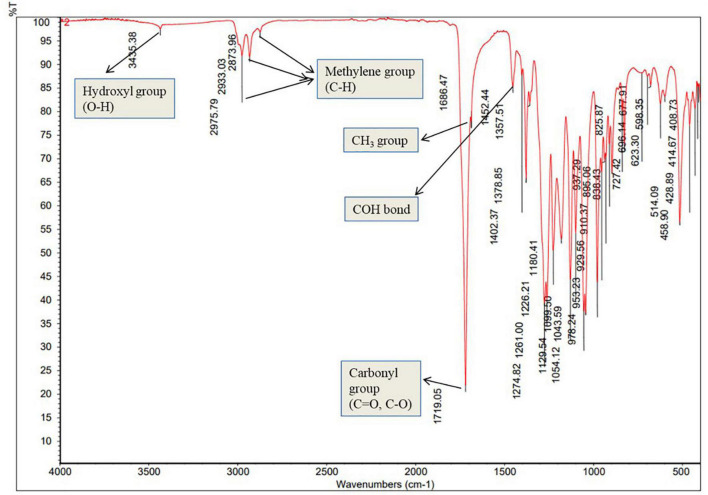
Fourier transform infrared analysis of polyhydroxybutyrate polymer extracted from strain RG36^T^ grown in medium containing glucose as carbon source.

The extracted PHB samples were evaluated for identification of their functional groups through FTIR analysis (Figure 5). The peak at 3,435.38 cm^–1^ correspond to the hydroxyl (O-H) group of alcohol, whereas the peak at 2,975.79, 2,933.03, and 2,873.96 cm^–1^ are due to methylene (C-H) stretch of alkanes. The significant peak at 1,719.05 cm^–1^ represents the carbonyl (C = O and C-O) stretch of ester. Furthermore, the peak at 1,452.44 cm^–1^ corresponds to CH showing asymmetrical stretching and bending vibration in CH_3_ group, whereas the peak at 1,357.51 represents the COH bond (Figure 6). The absorption band obtained from selected strain RG36^T^ was revealed to have similar peaks to standard (Mostafa et al., 2020), whereas the remaining peaks are closely lying between 3,430 and 400 cm^–1^. These all prominent absorption bands confirm the structure of PHB. The results are in agreement with the studies of other research groups working on PHB extracted from *Bacillus thuringiensis* (Alarfaj et al., 2015).

### ^1^H NMR and ^13^C NMR analysis

**FIGURE 7:**
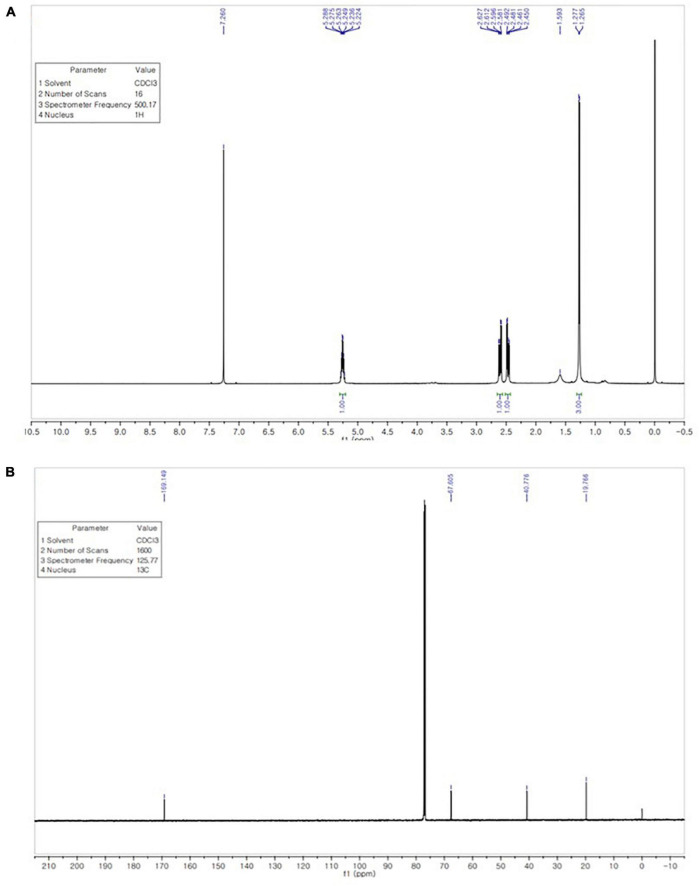
^1^H NMR spectrum shows signals at chemical shift δ 1.265–1.593 ppm (CH3), 2.450–2.627 ppm (CH_2_), and 5.224–5.288 ppm (CH) **(A)**. ^13^C NMR spectrum shows signals at 169.149, 67.605, 40.776, and 19.766 ppm for the carbon atom of the CO, CH, CH_2_, and CH_3_ groups **(B)**.

The ^1^H NMR spectrum of the extracted PHB in (Figure 7A) revealed signals of a methyl group at chemical shift δ 1.265–1.593 ppm. The pair of quadruplets of a methylene group attached to a carbonyl group (–CH_2_) was observed at δ = 2.450–2.627 ppm. Multiple signals appearing at δ = 5.224–5.288 ppm corresponded to a methine group (–CH). Another signal is observed at 7.260 ppm, attributed to the chloroform solvent.

The ^13^C NMR spectrum was analyzed to confirm the structure of the extracted biopolymer. The signal of the quaternary carbon of the carbonyl group was detected at δ 169.149 ppm, while the signal of the CH group was observed at δ 67.605 ppm. A intense signal at δ 40.776 ppm, the signal for the CH_2_ group appeared close to the carboxyl group, whereas the signal of the carbon of the methyl group was observed at δ 19.766 ppm (Figure 7B). The ^1^H NMR and ^13^C NMR spectra of extracted PHB from strain RG36^T^ was consistent with those reported in previous studies (Alarfaj et al., 2015; Mostafa et al., 2020).

### GC-MS analysis of extracted PHB

The PHB polymer isolated from strain RG36^T^ was analyzed by GC-MS to determine its monomeric composition of PHB (Supplementary Figure 2 and Supplementary Table 2). Three peaks with RT 6.12, 7.68, and 7.78 min were detected corresponding to hexanoic acid and 2-methyl-3- oxo-, ethyl ester (CAS), whereas another peak at RT 8.58 related to butanoic acid and 3-hydroxy-3-methyl. Additionally, a peak with RT of 8.8 and 16.57 for 2-butanoic acid, crotonic acid and for 2-butanoic acid and 1-methylethyl ester were also detected. These results are in agreement with the previous studies (Thirumala et al., 2010; Mostafa et al., 2020).

### Photodegradation of PHB in UV-light exposure and natural degradation of PHB in soil by microbes

**FIGURE 8:**
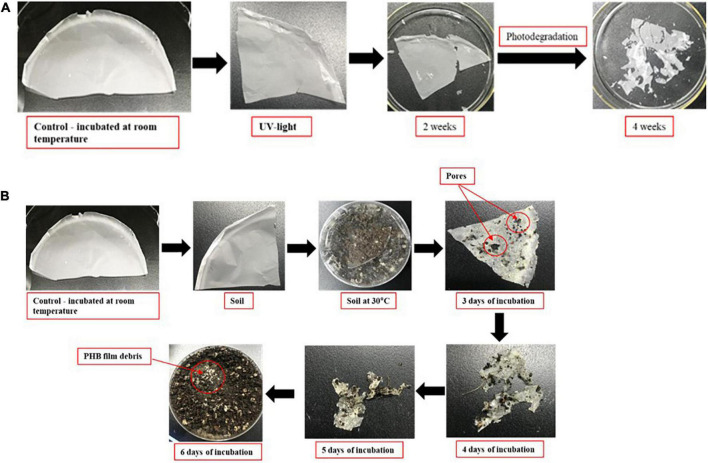
Degradation of PHB in UV-light exposure **(A)**. Degradation of PHB after 7 days of incubation at 30°C under soil collected from nearby field **(B)**.

After 2 weeks exposure of UV light, PHB film started to degrade slightly, progressing to intense photodegradation. The film appeared to be very thin (Figure 8A). The PHB surface became cracked, crushed, and become slightly rougher with increasing UV-light exposure. When incubated in soil, the PHB film showed a morphological change after 3 days and complete degradation occurred after 7 days (Figure 8B). Previous studies showed that PHB is degraded into water and carbon dioxide by bacteria in very short time (Park et al., 2022). Our study also showed the similar result in which PHB film was completely degraded by bacteria in 1 week as compared to UV light exposure. Degradation of PHB film was faster in soil conditions than under UV-light exposure. Microbes in soil completely degraded the PHB film. While the PHB exposed to UV-light only showed structural and morphological changes after 4 weeks of incubation (Figure 8A). Only a small amount of white debris was present in the soil on day 7 (Figure 8B).

### SEM observation of PHB

**FIGURE 9:**
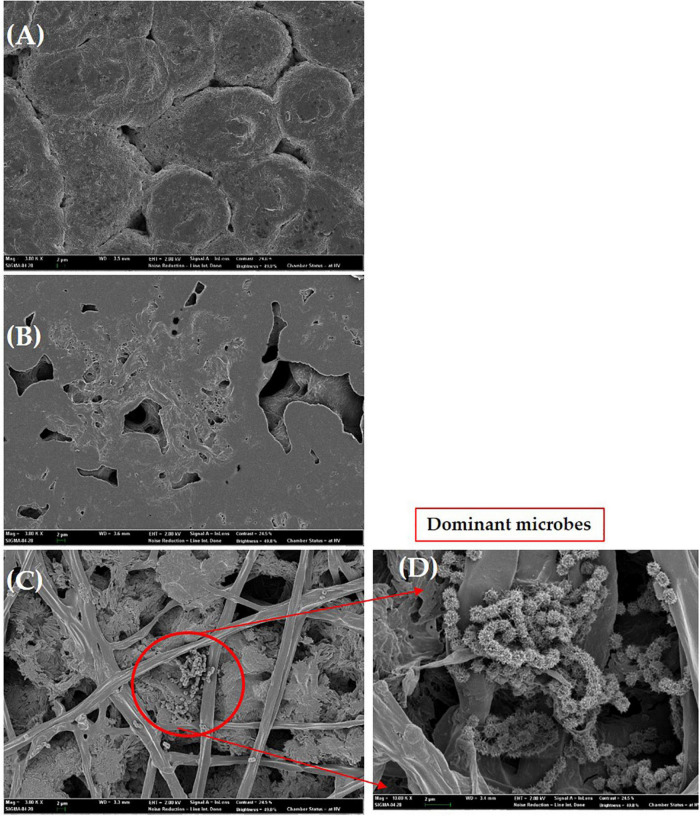
Scanning electron micrographs of the PHB film. Control **(A)**, UV exposure after 14 days **(B)**, degradation of PHB film in soil after 6 days of incubation **(C)**, dominant microbes similar to *Streptomyces* species attached to PHB film **(D)**. Bar **(A–D)**, 2 μm.

The PHB film was observed by SEM after 4 weeks of exposure to UV light. Several small and large holes and noticeable roughness, crests, and surface erosion were clearly observed on the PHB film compared to the control. Similar structural defects were seen for the PHB film incubated in soil which showed the presence of dominant microbes attached to the surface of the PHB film. The morphology of the microbes was found to be similar to *Streptomyces* species (Figures 9A–D). This species could secrete extracellular depolymerase enzyme responsible for the degradation of Supplementary Figure 3, showed the similar degradation results of PHB films at high magnifications (10.00 K X) (Supplementary Figures 3A–D). Presence of large and small holes, significant roughness, crests (Supplementary Figures 3A, B), presence of PHB fiber (Supplementary Figures 3C, D) with holes were found.

### Genomic features of strain RG36^T^

**FIGURE 10:**
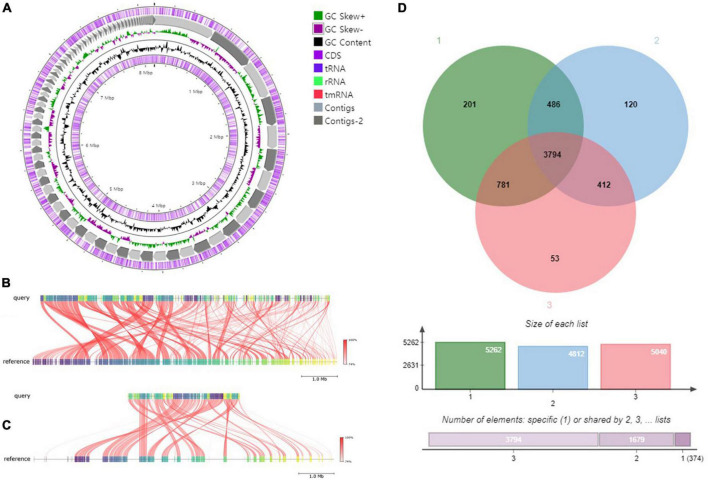
Graphical genome map of strain RG36^T^. RNA genes (tRNAs dark blue, rRNAs light green, and tmRNA red), GC content, and GC skew **(A)**. Illustration of FastANI’s work-flow for computing ANI between a query genome (strain RG36^T^) and a reference genome (*P. acidiphila* 7Q-K02^T^) **(B)** and *P. sacchari* IPT101^T^ **(C)**. Each red line segment denotes a reciprocal mapping between the query and reference genome, indicating their evolutionary conserved regions. Venn diagram of whole-genome orthologous genes in RG36^T^ and two reference strains. The numbers in the diagram indicate overlapped conserved genes or non-overlapped unique genes in each species. Strain: 1, RG36^T^; 2, *P. acidiphila* 7Q-K02^T^; 2, *P. sacchari* IPT101^T^ **(D)**.

The draft genome of strain RG36^T^ contained 119 contigs, with a total length of 8.52 Mb and an N50 length of 147,954 bp. A total of 7,702 genes were predicted, which included 7,381 protein-coding genes and 60 tRNA genes. The CheckM result showed that the genome completeness was 99% and the contamination level was 0.75%. Genomic circular feature map was constructed using CGView server is shown in Figure 10A. The ANI values between strain RG36^T^ and its two reference strains *P. acidiphila* 7Q-K02^T^ and *P. sacchari* IPT101^T^ were 84.2 and 88.6%, which are lower than the 95–96% cut-off values previously proposed for species delimitation (Jain et al., 2018). Each red line segment denotes a reciprocal mapping between the strain RG36^T^ and reference genomes, indicating their evolutionary conserved regions (Figures 10B, C). The dDDH relatedness values were 28.9 and 37%, which is lower than the 70% proposed threshold for the for species delineation (Meier-Kolthoff et al., 2013). The phylogenomic tree was well resolved and showed that *P. acidiphila* 7Q-K02^T^ and *P. sacchari* IPT101^T^ were the closest phylogenetic neighbor strains of strain RG36^T^ (Figure 1B). This result was consistent with the other phylogenetic tree (NJ) constructed by using 16S rRNA sequence. Previous study suggested that high number of contigs reduce the quality of bacterial genome sequence, which leads to poor assembly and unresolved the plasmid sequence (Smits, 2019). In this study, strain RG36^T^ revealed 119 contigs, which is very high. And might be due to poor assembly plasmid is not found in the genome of novel strain which is consistent with the previous reports mentioned above. The set of clusters of orthologous genes of strain RG36^T^ taken from the genome dataset, obtained using the eggNOG pipeline, assigned a total of 7,242 genes to 24 functional categories. Among the obtained functional groups, the cluster for [S] (function unknown; 2,647 genes) constituted the largest functional group (Figure 3). Among the other functional groups, the clusters for transcription [K] (transcription; 555), [E] (amino acid transport and metabolism; 539 genes), [C] (energy production and conversion; 532 genes), [G] (carbohydrate transport and metabolism; 412), [L] (replication, recombination, and repair; 351), [M] (cell wall/membrane/envelop biogenesis; 344), [P] (inorganic ion transport and metabolism; 313), [T] (signal transduction mechanisms; 262), [I] (lipid transport and metabolism; 233), [O] (post-translational modification, protein turnover, chaperons; 191), [J] (translation, ribosomal structure, and biogenesis; 175 genes), [H] (coenzyme transport and metabolism; 163), [Q] (secondary metabolites biosynthesis, transport, and catabolism; 150), [U] (intracellular trafficking, secretion, and vesicular transport; 120), [F] nucleotide transport and metabolism; 107), [V] (defense mechanism; 59), [N] cell motility; 55), [D] (cell cycle control, cell division, and chromosome partitioning; 38), [W] (extracellular structures; 4), [A] (RNA processing and modification; 1), and [B] (chromatin structure and dynamics; 1) were the represented categories (in descending order) (Supplementary Figure 4). The overall comparison analysis result is displayed as Venn diagram in Figure 10D. A total of 3,794 orthologous genes were shared among all three compared species, of which 486 were shared only between strain RG36^T^ and *P. acidiphila* 7Q-K02^T^ and 781 between strain RG36^T^ and *P. sacchari* IPT101^T^. The result of antiSMASH revealed that strain RG36^T^ encodes 14 gene clusters involved in secondary metabolite synthesis. Among them, there were five gene clusters for terpene, two for RRE-element, one for phosphonate, NRPS-independent-siderophore, ladderane, RiPP-like, hserlactone, arylpolyene, and redox-cofactor.

### Genes involved in plant growth promotion

A thorough genomic analysis of strain RG36^T^ revealed the presence of genes that have been involved in promoting plant growth. A diverse group of PGP bacteria is able to reduce plant ethylene levels by the action of 1-aminocyclopropane-1-carboxylate (ACC) deaminase (Chhetri et al., 2022a). Genome annotation of strain RG36^T^ revealed a gene cluster for ACC, a coding enzyme, which can degrade the ethylene precursor ACC, and decrease the ethylene levels in plants. Strain RG36^T^ harbored only one gene related to nitrogen fixation *nifU*, the other sets of nitrogen-fixation genes were not found. This is could be due to the gaps in the draft genome sequence of strain RG36^T^. A set of genes for IAA and siderophore production were also found, which is consistent with our result above. Genome annotation by NCBI revealed a full set of genes responsible for phosphate solubilization and conferred the ability of strain RG36^T^ to solubilize insoluble phosphate in the PVK agar plate (Table 2). PSB release gluconic acid that is synthesized by the interaction between co-factor pyrroloquinoline quinine (PQQ) and glucose dehydrogenase within themselves and hence convert the insoluble phosphate into a soluble form (Ahemad and Kibret, 2014). A total of five gene products, *PqqA*, *PqqB*, *PqqC*, *PqqD*, and *PqqE*, are identified in the genome of strain RG36^T^ for PQQ production (Table 3). Other genes responsible for reduction of ACC, nitrogen fixation, indole acetic acid, and siderophore production are also revealed and presented (Table 4).

**TABLE 2:** Putative genes involved in phosphate solubilization in the genome of strain RG36^T^.

Function	Locus tag	Annotation
Phosphate solubilization	L5014_02050	Phosphonoacetate hydrolase **(*phnA*)**
	L5014_13070	Phosphonate metabolism transcriptional regulator **(*phnF*)**
	L5014_03125	Phosphonate ABC transporter, permease protein **(*phnE*)**
	L5014_13060	Phosphonate metabolism protein/1,5-bisphosphokinase (PRPP-forming) **(*phnN*)**
	L5014_34695	2-aminoethylphosphonate ABC transporter substrate-binding protein **(*phnS*)**
	L5014_34700	2-aminoethylphosphonate ABC transport system ATP-binding subunit **(*phnT*)**
	L5014_34710	2-aminoethylphosphonate ABC transport system, membrane component **(*phnV*)**
	L5014_02055	Phosphonoacetaldehyde dehydrogenase **(*phnY*)**
	L5014_15030	Exopolyphosphatase **(*ppx*)**
	L5014_15025	Polyphosphate kinase 1 **(*ppk1*)**
	L5014_15015	Phosphate regulon transcriptional regulator **(*phoB*)**
	L5014_15020	Phosphate regulon sensor histidine kinase **(*phoR*)**
	L5014_15010	Phosphate signaling complex protein **(*phoU*)**
	L5014_15000	Phosphate ABC transporter permease **(*pstA*)**
	L5014_15005	Phosphate ABC transporter ATP-binding protein **(*pstB*)**
	L5014_14995	Phosphate ABC transporter permease **(*pstC*)**
	L5014_14990	Phosphate ABC transporter substrate-binding protein **(*pstS*)**
	L5014_19095	Glucose-6-phosphate dehydrogenase **(*zwf*)**
	L5014_19235	Phosphate acetyltransferase **(*pta*)**

Abbreviations of genes are highlighted in bold.

**TABLE 3:** Putative genes associated with pyrroloquinoline-quinone synthase in the genome of strain RG36^T^.

Function	Locus tag	Annotation
Pyrroloquinoline quinine	L5014_01040	Pyrroloquinoline-quinone synthase **(*PqqC*)**
	L5014_27155	Pyrroloquinoline-quinone synthase **(*PqqC*)**
	L5014_36850	Pyrroloquinoline-quinone synthase **(*PqqC*)**
	L5014_01030	Pyrroloquinoline quinone biosynthesis protein **(*PqqE*)**
	L5014_01045	Pyrroloquinoline quinone biosynthesis protein **(*PqqB*)**
	L5014_01050	Pyrroloquinoline quinone precursor peptide **(*PqqA*)**
	L5014_27160	Pyrroloquinoline quinone biosynthesis protein **(*PqqB*)**
	L5014_27165	Pyrroloquinoline quinone precursor peptide **(*PqqA*)**
	L5014_36855	Pyrroloquinoline quinone biosynthesis protein **(*PqqB*)**
	L5014_36860	Pyrroloquinoline quinone precursor peptide **(*PqqA*)**
	L5014_38330	Pyrroloquinoline quinone biosynthesis protein **(*PqqE*)**, partial
	L5014_01035	Pyrroloquinoline quinone biosynthesis peptide chaperone **(*PqqD*)**
	L5014_27150	Pyrroloquinoline quinone biosynthesis peptide chaperone **(*PqqD*)**
	L5014_36845	Pyrroloquinoline quinone biosynthesis peptide chaperone **(*PqqD*)**

Abbreviations of genes are highlighted in bold.

**TABLE 4:** Putative genes involved in the PGP in the genome of strain RG36^T^.

Function	Locus tag	Annotation
Reduction of ACC	L5014_15340	1-aminocyclopropane-1-carboxylate deaminase
Nitrogen fixation	L5014_04090	SUF system NifU family Fe-S cluster assembly protein
Production of IAA	L5014_35350	Anthranilate phosphoribosyltransferase **(*trpD*)**
	L5014_35340	Anthranilate synthase component I **(*trpE*)**
	L5014_35345	Aminodeoxychorismate/anthranilate synthase component II
	L5014_16110	Anthranilate 1,2-dioxygenase large subunit **(*antA*)**
	L5014_16115	Anthranilate 1,2-dioxygenase small subunit **(*antB*)**
	L5014_16120	Anthranilate 1,2-dioxygenase electron transfer component **(*antC*)**
	L5014_35355	Indole-3-glycerol phosphate synthase **(*trpC*)**
	L5014_01255	Tryptophan 7-halogenase
	L5014_08200	Tryptophan 7-halogenase
	L5014_08870	Tryptophan–tRNA ligase
	L5014_35730	Tryptophan leader peptide
	L5014_36295	Tryptophan 7-halogenase
	L5014_19995	Tryptophan 2,3-dioxygenase **(*kynA*)**
	L5014_15515	Tryptophan synthase subunit beta **(*trpB*)**
	L5014_15525	Tryptophan synthase subunit alpha **(*trpA*)**
Production of siderophore	L5014_04130	Siderophore-interacting protein
	L5014_18970	TonB-dependent siderophore receptor
	L5014_26620	Siderophore ferric iron reductase
	L5014_26625	IucA/IucC family siderophore biosynthesis protein

Abbreviations of genes are highlighted in bold.

Microbes can either flow through soil water fluxes toward the roots, or actively induce flagellar activity enabling internalization and colonization of different parts of the plant (Compant et al., 2009; Osman et al., 2017). TEM visualization of strain RG36^T^ also revealed the presence of single flagellum and genome analysis revealed genes for flagellar biosynthesis, assembly, and chemotaxis (Supplementary Table 3). Draft genome sequence of strain RG36^T^ also revealed other gene clusters associated with quorum sensing and secretion systems. The secretion system translocates bacterial effector molecules into host cells. Moreover a set of genes involve in quorum sensing were also found which play important roles in the host colonization and establishment of strain RG36^T^. Quorum-sensing autoinducers, such as N-acyl-homoserine lactones (AHL), cause a pronounced interkingdom signaling effect on plants, provoking priming processes of pathogen defense and insect pest control (Issa et al., 2018; Kuramae et al., 2020). In addition, some PGP bacteria only harbor genes for AHL receptors, so-called LuxR-solo genes, which can contribute to plant growth promotion and biological control (Kuramae et al., 2020). In our study, we also found only LuxR genes in the genome of strain RG36^T^. The list of genes are presented in Supplementary Table 4. Bacteria possess protein secretion systems that play important roles in biotic interactions, such as colonization and pathogenicity (Iniguez et al., 2005). The genome of strain RG36^T^ possesses genes for type I, II, III, IV, and VI systems, however, the genes for type V were not found. Type I system is responsible for the export of small molecules, transfer of molecules to recipient cells, and secretion of unfolded proteins, type II which is responsible for the secretion of folded proteins. Strain RG36^T^ harbors only four genes for the type III secretion system, which are known to be large nanomachines, utilized by both pathogenic and symbiotic bacteria to inject effector proteins directly into the cytoplasm of host cells and facilitate host cell colonization (Kuramae et al., 2020; Tapia-García et al., 2020). Type IV, responsible for the secretion of large molecules into the cytoplasm of recipient cells. Type V, which is responsible for the secretion of virulence factors, were not found. Compared to other secretion systems, genes encoding the type VI secretion system were overrepresented in the genome of strain RG36^T^ (Supplementary Table 5). A previous study showed that T6SSs participate in antibacterial activity, biofilm formation metal ion uptake, such as that of iron, manganese, and zinc (Gallique et al., 2017).

### Genes related to PHB synthesis

Polyhydroxybutyrates synthases (*PhaC*) and phasins (*PhaP*) are proteins that play important roles in PHB production and granule formation. Phasin, forms a boundary layer on the PHB surface to sequester hydrophobic PHB from the cytoplasm (Pötter et al., 2002). The set of genes for PHB synthase were found in the genome of RG36^T^: *phaC*, *phaZ*, *phaP*, and *phbB* were found (Table 5). Expression of the *phaR* gene is negatively controlled by the autoregulated repressor *phaR* in *Ralstonia eutropha* (Urtuvia et al., 2018), which is also found in the genome of strain RG36^T^.

**TABLE 5:** Annotation of genes responsible for PHB synthesis in strain RG36^T^.

Accession	Start	Stop	Locus tag	Length	Protein name
JAKLJA010000006	93,066	95,003	L5014_10540	645	Class I poly(R)-hydroxyalkanoic acid synthase **(*phaC*)**
JAKLJA010000026	22,809	24,536	L5014_25360	575	Class I poly(R)-hydroxyalkanoic acid synthase **(*phaC*)**
JAKLJA010000018	69,278	70,555	L5014_20810	425	Polyhydroxyalkanoate depolymerase **(*phaZ*)**
JAKLJA010000013	68,557	69,117	L5014_17330	186	TIGR01841 family phasin **(*phaP*)**
JAKLJA010000020	54,712	55,272	L5014_21885	186	TIGR01841 family phasin **(*phaP*)**
JAKLJA010000048	16,987	17,481	L5014_32705	164	TIGR01841 family phasin **(*phaP*)**
JAKLJA010000093	4,843	5,439	L5014_38120	198	TIGR01841 family phasin **(*phaP*)**
JAKLJA010000006	89,991	90,602	L5014_10525	203	Polyhydroxyalkanoate synthesis repressor **(*phaR*)**
JAKLJA010000007	195,750	196,493	L5014_12160	247	Acetoacetyl-CoA reductase **(*phbB*)**
JAKLJA010000026	54,246	54,989	L5014_25520	247	Acetoacetyl-CoA reductase **(*phbB*)**
JAKLJA010000048	14,554	15,297	L5014_32695	247	Acetoacetyl-CoA reductase **(*phbB*)**
JAKLJA010000093	5,639	6,382	L5014_38125	247	Acetoacetyl-CoA reductase **(*phbB*)**

Abbreviations of genes are highlighted in bold.

## Conclusion

There have been no previous studies on the diversity of bacteria in marigold roots. Hence, this study was conducted, to explore the diversity of culturable bacteria in the roots of marigolds growing near Dongguk University, Ilsan, Republic of Korea. After screening, species of the genus *Paraburkholderia*, strain RG36^T^ was selected for this study. 16S rRNA gene phylogeny, genome sequence comparison, and physiological results showed that strain RG36^T^ represent a novel species of genus *Paraburkholderia*. The ANI and dDDH values between the strain RG36^T^ and the two closest related species *P. acidiphila* 7Q-K02^T^ and *P. sacchari* IPT101^T^ were found to be 84.2/88.6 and 28.9/37%, respectively, which support the delineation of new species. The result of biochemical and genomic functional analyses showed that strain RG36^T^ is a novel PHB producing bacteria. Most strains in the genus *Paraburkholderia* exhibit a common ability to produce PHB. Since the species is belongs to the genus *Paraburkholderia*, which are already popular for bioplastic production in good amount, so the production of bioplastic from the novel species of *Paraburkholderia*, strain RG36^T^ was also investigated in this study by using glucose as a carbon source. Intracellular PHB granules were directly detected by Sudan black B staining procedure and confirmed by microscopic observations. The optimum pH, temperature, and incubation period for the highest PHB production by the isolate were pH 7, 30°C and 72 h, respectively. The PHB extracted from strain RG36^T^ was characterized using FTIR, ^1^H NMR, ^13^C NMR analysis, and GC-MS analysis. Genomic annotation of strain RG36^T^ revealed a set of genes involved in PHA and PHB production: pha*C* (2, gene cluster), pha*Z* (1), pha*P* (4), pha*R* (1), and phb*B* (4) which are already known for PHB production.

Furthermore, to identify the potential of strain RG36^T^ in agriculture, we investigated the PGP effect of strain RG36^T^ on tomato plants. Production of floating biofilm suggest that *Paraburkholderia* sp. RG36^T^ possesses the ability to colonize and promote the growth of tomato plants. Genome sequence of strain RG36^T^ provided insights into its plant growth promotion, biocontrol activities, and defense capabilities. NCBI annotation revealed the presence of siderophore gene clusters and genes involved IAA production, phosphate solubilization, ACC, and nitrogen fixation, which are all associated with plant growth promotion and biocontrol of strain RG36^T^. The genome information and *in vitro* results obtained in this study provides more clues for the potential application of the strain in the development of eco-friendly bio-fertilizers, which can promote soil fertility and crop yield.

Previous study showed, *Burkholderia seminalis* strain 869T2 was shown to synthesize 2.0–2.2 μg/ml of IAA in the presence of tryptophan and increased both the shoot and root biomass of tested plant tissues (Hwang et al., 2021). In comparison, our strain RG36^T^ produced 12.5 μg/ml IAA in the presence of L-tryptophan which is higher. IAA is a naturally occurring auxin produced by several endophytic bacterial species through the L-tryptophan metabolism pathway. Tryptophan is present in the root exudates of plants and is utilized by the bacteria to synthesize auxin, which enhances the growth of host plants (Weselowski et al., 2016; Suárez-Moreno et al., 2019). Auxin is the major plant hormone that regulates various aspects of plant growth and development, such as root initiation and development, fruit development, leaf formation, floral initiation and patterning, phototropism, and embryogenesis (Kou et al., 2022). Another study showed that *Paenibacillus polymyxa* strain CR1 isolated from degrading corn roots produced IAA, display antagonistic activities against common plant pathogens and improved growth of maize, potato, cucumber, tomato, and Arabidopsis plants upon inoculation (Weselowski et al., 2016).

We have documented the IAA production, siderophore synthesis, nitrogen fixation, and phosphate solubilization abilities of strain RG36^T^. Inoculations of strain RG36^T^ into tomato plants demonstrated the plant growth promotion ability of this bacterium. The fresh and dried, shoot, and root weights of tomato plants were significantly increased. There was a significant increase in length of roots as compared to control. Most importantly, strain RG36^T^ found to increase the yield of tomatoes, which could be a major advantage for farmers. As we already mentioned in introduction about previous studies that showed that colonization of PHB in plant root, notably increased root area and deletion of PHB related genes decreased the PGP ability of bacteria. So in our study also, PHB could be one of the reason for increased root length of tomato and other PGP activity of strain RG36^T^. We also detected the presence of PHB related genes that had been deleted in previous studies, and their deletion decreased the PGP ability of bacteria. Our results suggest that the IAA synthesis, siderophore production, nitrogen fixation, ACC reduction, and phosphate solubilization abilities of *Paraburkholderia* species RG36^T^ may collectively contribute to the growth enhancement observed in tomato plant tested here.

At last, this study also reveals that marigold roots from where the strain RG36^T^ was isolated is a potent ecological niche with having inimitable strain diversity which are yet to be discovered, especially in Republic of Korea. Therefore, this unique undiscovered habitats should be continuously explored for extraction of beneficial microbes and deserve study in depth.

The current investigation demonstrates that the roots of marigold plant are a potential source for beneficial bacterial isolates. Strain RG36^T^ can be considered as good candidates for industrial production of PHB from glucose, which can reduce environmental pollution problems caused by conventional plastics and solve disposal problem of the agricultural wastes. However, optimization is required by using more carbon and nitrogen sources at different concentrations, temperatures, and pH for good production of PHB by strain RG36^T^.

### Description of *Paraburkholderia tagetis* sp. nov.

*Paraburkholderia tagetis* sp. nov. (ta.ge’tis. N.L. gen. n. *tagetis*, of *Tagetes*, the plant from which the type strain was isolated).

Cells of are Gram-stain negative, motile by one polar flagellum, aerobic and rod-shaped, with dimensions of 0.6–1.0 μm in width and 1.3–2.5 μm in length. Colonies are white, convex, thick, and smooth with entire margins in R2A agar plates. Growth occurs at 4–35°C (optimum, 28–30°C), pH 5.5–9.0 (pH 6.5–7.5) and in the presence of 0–3% (w/v) NaCl (0%, w/v). Strain RG36^T^ is positive for catalase and oxidase activities. Grew well in R2A, NA, and TSA, however, no growth was occurred in MA and LB agar plates. In API 20NE, nitrate is not reduced. Strain is positive for glucose fermentation, indole production, and arginine dihydrolase. Assimilation of L-arabinose, D-mannose, D-mannitol, potassium gluconate, adipic acid, malic acid, and phenylacetic acid is occurred, however, β-galactosidase, D-glucose, N-acetyl-D-glucosamine, capric acid, trisodium citrate are not. It can hydrolyze esculin, gelatin and casein but does not hydrolyze CM cellulose, Tween 80, and chitin. In API ZYM, the type strain is positive for alkaline phosphatase, esterase (C4), esterase lipase (C8), lipase (C14), leucine arylamidase, α-chymotrypsin, trypsin, acid phosphatase, N-acetyl-β-glucosaminidase, α-mannosidase, and α-galactosidase but negative for valine arylamidase, cystine arylamidase, Naphthol-AS-BI-phosphohydrolase, β-galactosidase, β-glucosidase, β-glucuronidase, α-glucosidase, β-glucosidase, and α-fucosidase. The predominant respiratory quinone is Q-8. The principal cellular fatty acids are C_16:0_, C_17:0_ cyclo, C_19:0_ cyclo ω8c, summed feature 8 (comprising C_18:1_ ω7c and/or C_18:1_ ω6c). The DNA G + C content of the type strain is 63.7%.

The type strain, RG36^T^ (=KACC 22685^T^ = TBRC 15696^T^), was isolated from rhizosphere of marigold plant, geographically located at Ilsan, Gyeonggi-do, Republic of Korea (37° 40′ 26.4″ N 126° 48′ 20.88″ E). The GenBank/EMBL/DDBJ accession numbers for the 16S rRNA gene sequence and the whole-genome sequence of strain RG36^T^ are OL347860 and JAKLJA000000000, respectively.

## Data availability statement

The datasets presented in this study can be found in online repositories. The names of the repository/repositories and accession number(s) can be found in the article/Supplementary material.

## Author contributions

GC designed the research, analyzed the data, and prepared the manuscript. IK carried out the experiments, advised on the experimental design, and English language editing. JK and YS performed the pot experiments. SP and YJ collected the samples and analyzed the sequencing data. TS coordinated and supervised the study. All authors read and agreed to the published version of the manuscript.

## Footnotes

1
https://lpsn.dsmz.de/genus/paraburkholderia

2
https://ecogenomics.github.io/CheckM
