# Supplementary material for: Paraburkholderia tagetis sp. nov., a novel species isolated from roots of Tagetes patula enhances the growth and yield of Solanum lycopersicum L. (tomato)
Source: Front Microbiol. 2023 Apr 4;14:1140484. doi: 10.3389/fmicb.2023.1140484 (PMC10110911; doi:10.3389/fmicb.2023.1140484)

***Paraburkholderia tagetis* sp. nov., a novel species isolated from roots of *Tagetis patula* enhances the growth and yield of *Solanum lycopersicum* L. (Tomato)**

Author names

Geeta Chhetri, Inhyup Kim, Jiyoun Kim, Yoonseop So, Sunho Park, Yonghee Jung and Taegun Seo*

**Affiliation**

Department of Life Science, Dongguk University-Seoul, Goyang 10326, South Korea

*** Corresponding Author:**

Dr. Taegun Seo

[tseo@dongguk.edu](mailto:tseo@dongguk.edu)

Tel : +82-31-961-5135

**Supplementary Information**

**Summary:**

**Figure S1.** Transmission electron microscope image of a negatively stained cell of strain RG36^T^ cultivated at 30ºC for 4 days on R2A agar. Bar, 0.5 μm. (A), bacterial colonies showing clear halo zone around the microbial growth of novel phosphate dissolving bacterial isolate (B) and sudan stained black PHB granules of strain RG36^T^ under 1000X, with and without PHB granule (C).

**Figure S2.** GC-mass spectra of PHB extracted from strain RG36^T^. Three peaks with RT 6.12, 7.68 and 7.78 min were detected corresponding to hexanoic acid and 2- methyl-3-oxo-, ethyl ester (CAS) (A, B, C), whereas another peak at RT 8.58 related to butanoic acid and 3-hydroxy-3-methyl (D). Additionally, a peak with RT of 8.8 (E) and 16.57 for 2-butanoic acid, crotonic acid and for 2-butanoic acid and 1-methylethyl ester were also detected (F).

**Figure S3.** Morphological changes in SEM micrographs of PHB film degraded after four weeks in UV light exposure (A), (B) and in soil by microbes after six days of incubation (C), (D). Large number of similar microbes were found to attach on the surface of PHB (C), (D).

## Figure S4. Distribution of genes based on the 24 general eggNOG functional categories of strain RG36^T^.

**Table S1.** Cellular fatty acid profiles of strain RG36^T^ and closely related species. Strain: 1. RG36^T^; 2; *P. acidiphilla* 7Q-K02^T^; 3, *P. sacchari* IPT101^T^. All data were obtained from this study. Data are expressed as percentages of the total fatty acids, and fatty acids amounting to <1 % in all strains are not shown. Major components (>10.0 %) are highlighted in bold. TR, Trace amount (<1 %); –, not detected.

**Table S2.** GC-MS data table for PHB.

**Table S3.** Putative genes associated with chemotaxis and motility in the genome of strain RG36^T^.

**Table S4.** Putative genes associated quorum sensing in the genome of strain RG36^T^.

**Table S5.** Putative genes associated with secretion systems in the genome of strain RG36^T^.

**Figure S1.** Transmission electron microscope image of a negatively stained cell of strain RG36^T^ cultivated at 30ºC for 4 days on R2A agar. Bar, 0.5 μm. (A), bacterial colonies showing clear halo zone around the microbial growth of novel phosphate dissolving bacterial isolate (B) and sudan stained black PHB granules of strain RG36^T^ under 1000X, with and without PHB granule (C).


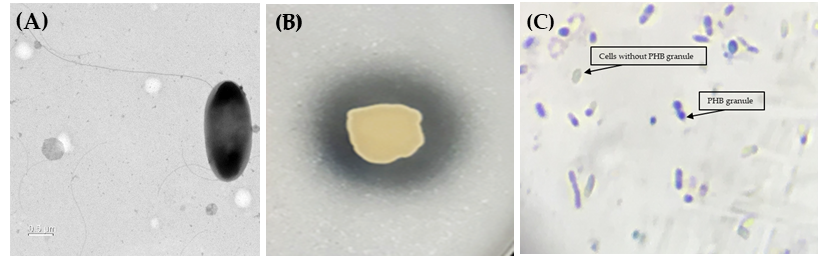


**Figure S2.** GC-mass spectra of PHB extracted from strain RG36^T^. Three peaks with RT 6.12, 7.68 and 7.78 min were detected corresponding to hexanoic acid and 2- methyl-3-oxo-, ethyl ester (CAS) (A, B, C), whereas another peak at RT 8.58 related to butanoic acid and 3-hydroxy-3-methyl (D). Additionally, a peak with RT of 8.8 (E) and 16.57 for 2-butanoic acid, crotonic acid and for 2-butanoic acid and 1-methylethyl ester were also detected (F).


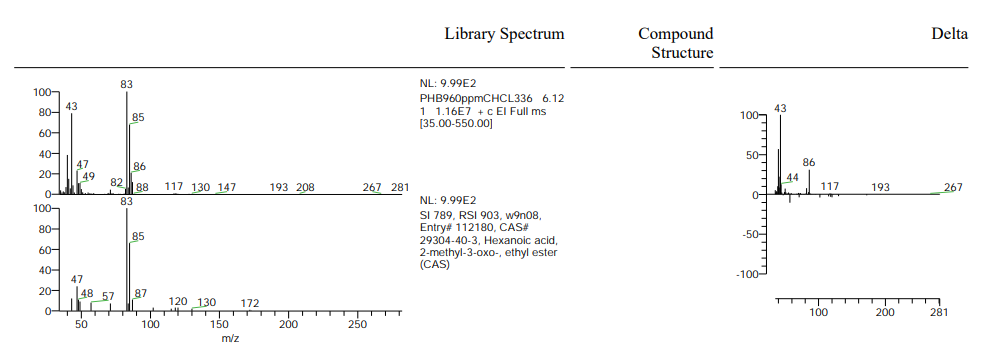


(A)


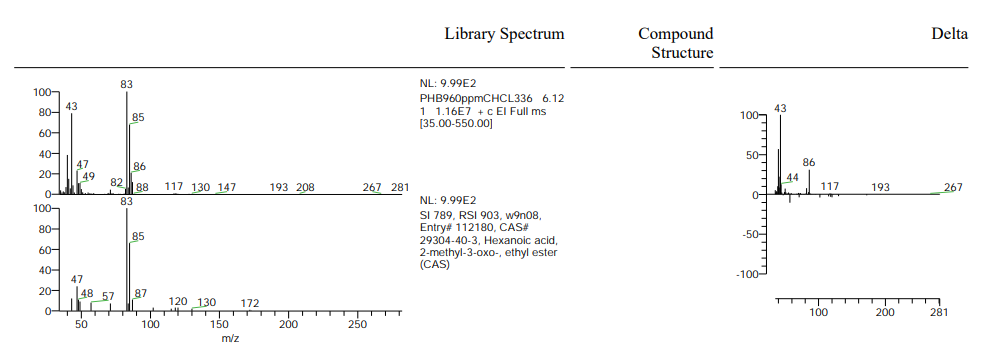


(B)


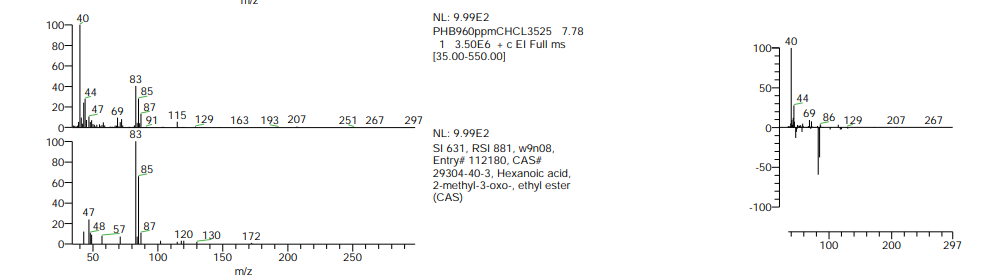


(C)


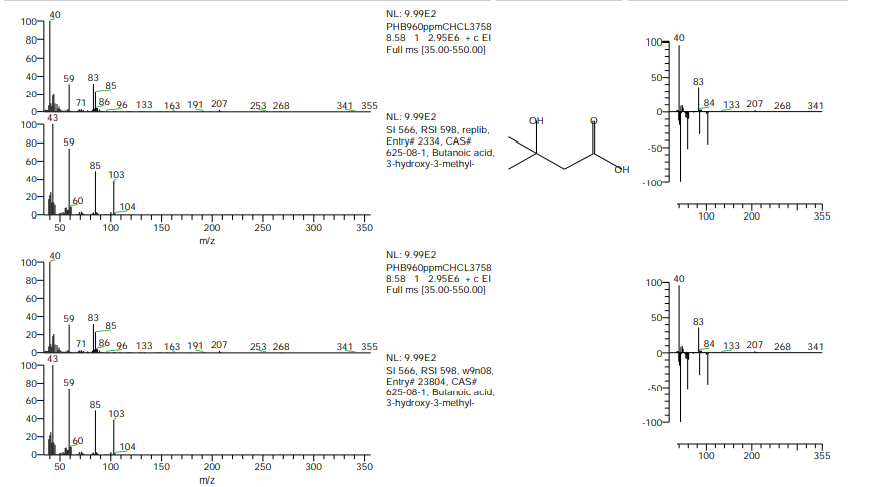


(D)


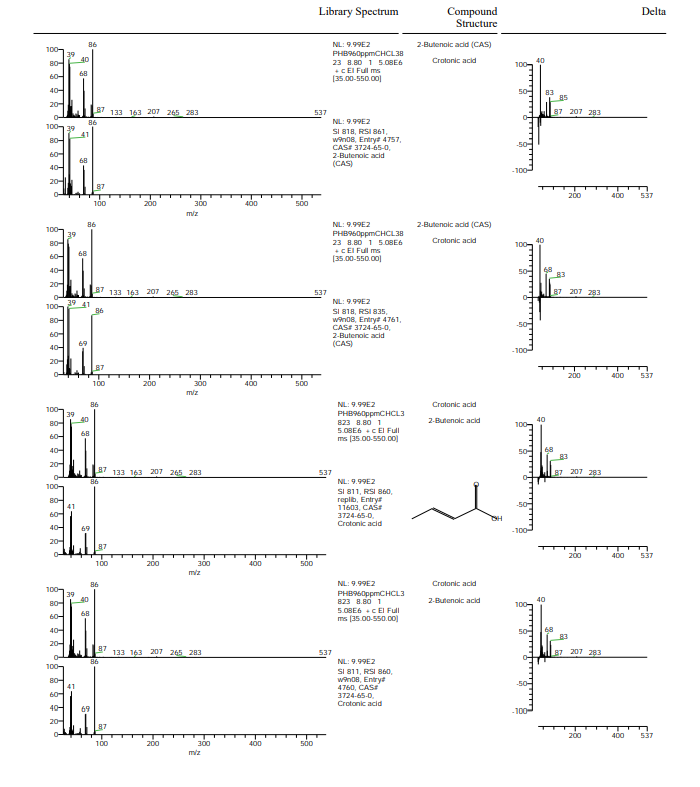


(E)


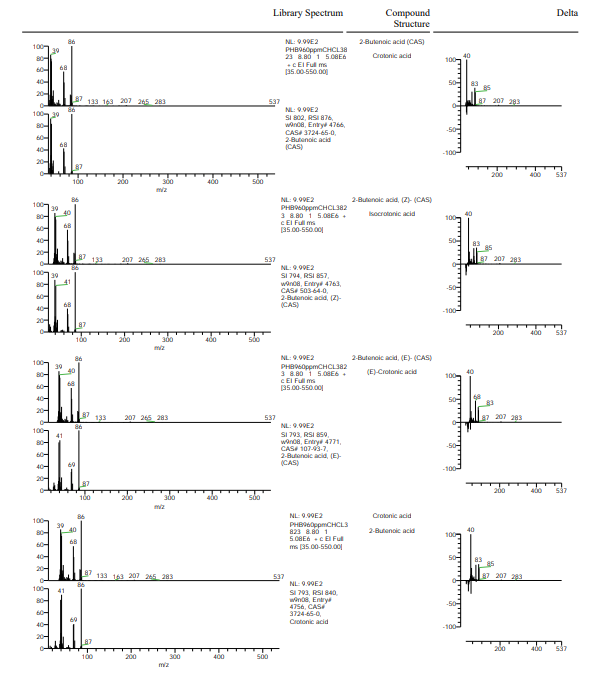


(E)


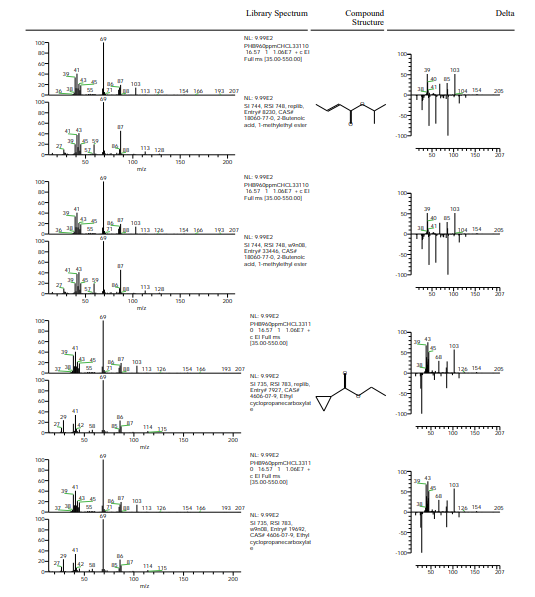


(F)

**Figure S3.** Morphological changes in SEM micrographs of PHB film degraded after four weeks in UV light exposure (A), (B) and in soil by microbes after six days of incubation (C), (D). Large number of similar microbes were found to attach on the surface of PHB (C), (D).


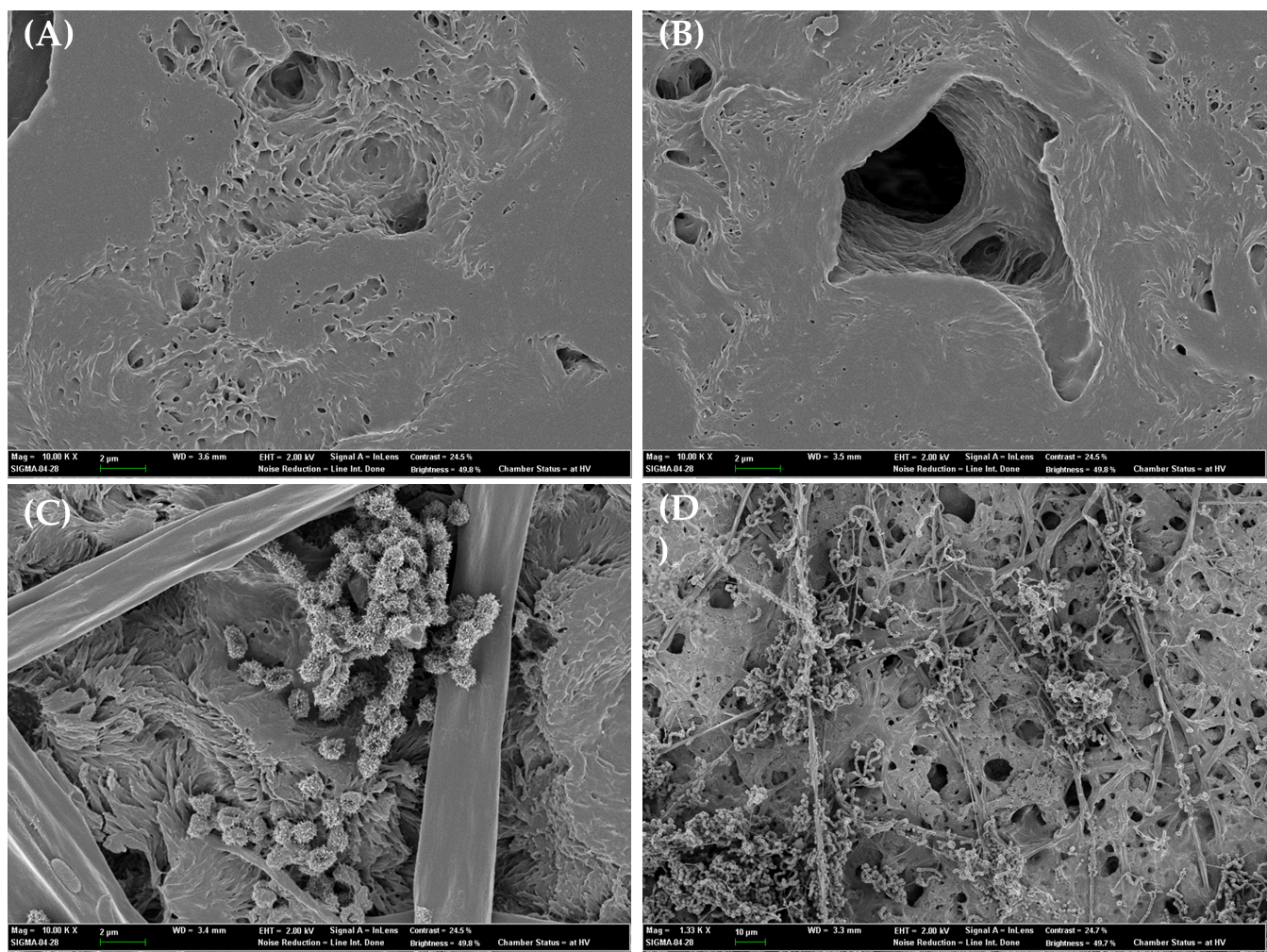


## Figure S4. Distribution of genes based on the 24 general eggNOG functional categories of strain RG36^T^.

##
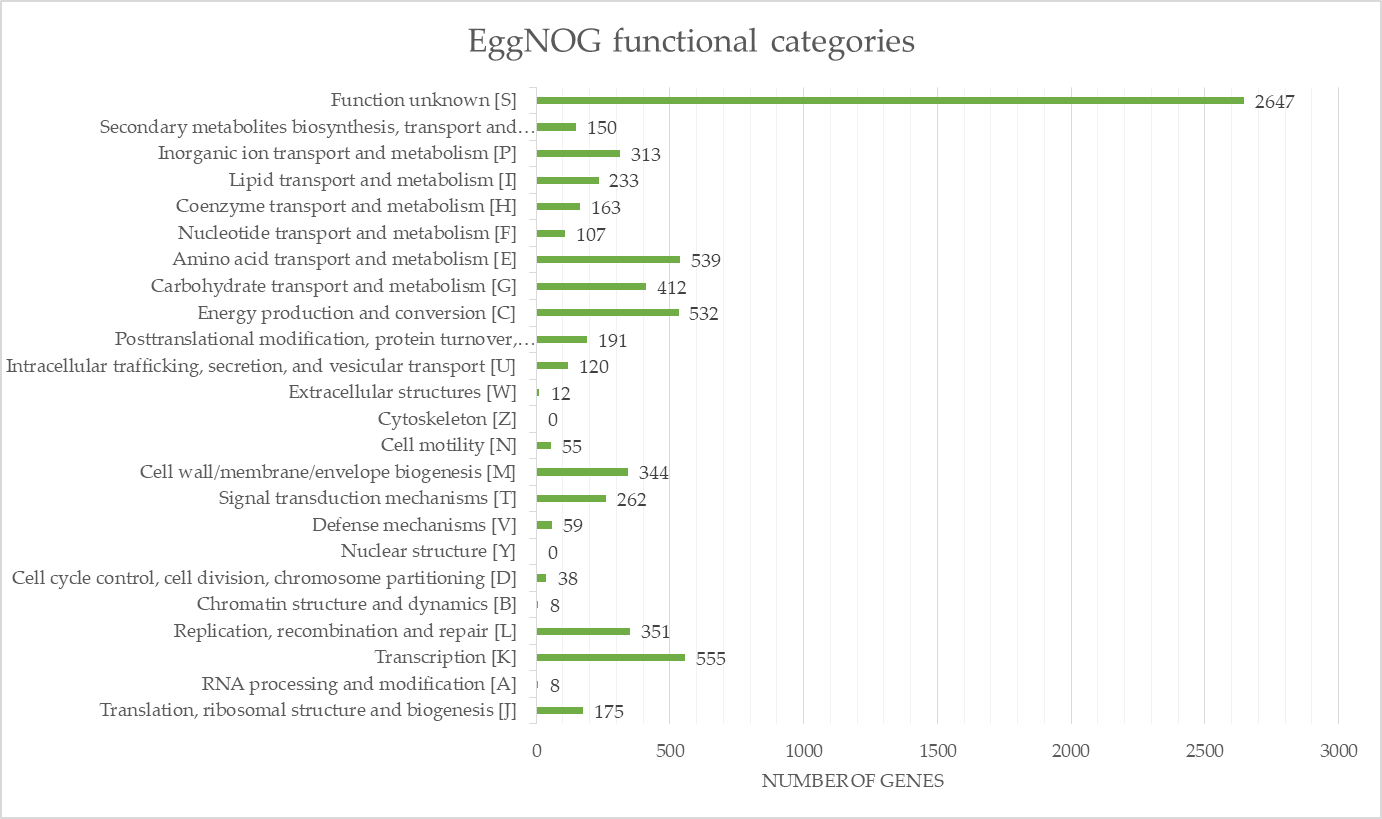


**Table S1.** Cellular fatty acid profiles of strain RG36^T^ and closely related species. Strain: 1. RG36^T^; 2; *P. acidiphilla* 7Q-K02^T^; 3, *P. sacchari* IPT101^T^. All data were obtained from this study. Data are expressed as percentages of the total fatty acids, and fatty acids amounting to <1 % in all strains are not shown. Major components (>10.0 %) are highlighted in bold. TR, Trace amount (<1 %); –, not detected.

| **Fatty acid** | **1** | **2** | **3** |
| --- | --- | --- | --- |
| C_14 : 0_ | 6.2 | 4.4 | TR |
| **C_16 : 0_** | **26.3** | **25.8** | **20.8** |
| C_16 : 0_ 2-OH | 3.2 | 2.4 | 6.6 |
| C_16 : 0_ 3-OH | 6.2 | 5.3 | 3.8 |
| C_16 : 1_ 2-OH | 1.6 | – | 1.4 |
| C_18 : 1_ 2-OH | TR | 1.3 | 2.9 |
| iso C17:0 3-OH | – | 1.8 | 1 |
| **C_17 : 0_ cyclo** | **23.5** | **22.1** | **21.9** |
| **C_19 : 0_ cyclo *ω*8*c*** | **10.6** | **16.3** | **19.8** |
| Summed features* |  |  |  |
| 2 | 6.2 | 2.9 | 5.8 |
| 3 | 2.8 | 3.1 | 2.1 |
| **8** | **13.4** | **14.2** | **13.4** |

*Summed features are fatty acids that cannot be resolved reliably from another fatty acid using the chromatographic conditions chosen. The MIDI system groups these fatty acids together as one feature with a single percentage of the total. Summed feature 2 comprises iso-C_16 : 1_I/C_14 : 0_3-OH, summed feature 3 comprises C_16 : 1_ ω7c/C_16 : 1_ ω6c and summed feature 8 comprises C_18 : 1_ ω7c and/or C_18 : 1_ ω6c.

**Table S2.** GC-MS data table for PHB.

| **RT** | **Compound name** | **Molecular formula** | **Molecular weight g/mol** |
| --- | --- | --- | --- |
| 6.12 | Hexanoic acid, 2-methyl-3-oxo-, ethyl ester | C_9_H_16_O_3_ | 172.2 |
| 7.68 | Hexanoic acid, 2-methyl-3-oxo-, ethylester (CAS) | C_9_H_16_O_3_ | 172.2 |
| 7.78 | Hexanoic acid, 2-methyl-4-oxo-, ethylester (CAS) | C_9_H_16_O_4_ | 172.2 |
| 8.58 | Butanoic acid, 3-hydroxy-3-methyl | C_5_H_10_O_3_ | 118.3 |
| 8.8 | 2-Butenoic acid, Crotonic acid, | C_4_H_6_O_2_ | 86.09 |
| 16.57 | 2-Butenoic acid, 1-methylethyl ester | C_7_H_12_O_2_ | 128.17 |

**Table S3.** Putative genes associated with chemotaxis and motility in the genome of strain RG36^T^.

| **Function** | **Locus tag** | **Annotation** |
| --- | --- | --- |
| Chemotaxis | L5014_17470 | chemotaxis protein |
|  | L5014_01375 | chemotaxis protein (*CheW*) |
|  | L5014_01385 | chemotaxis protein (*CheW*) |
|  | L5014_16595 | chemotaxis protein (*CheW*) |
|  | L5014_16610 | chemotaxis protein (*CheR*) |
|  | L5014_17615 | chemotaxis protein (*CheB*) |
|  | L5014_19440 | chemotaxis protein (*CheC*) |
|  | L5014_22260 | chemotaxis protein (*CheB*) |
|  | L5014_01370 | methyl-accepting chemotaxis protein |
|  | L5014_02260 | methyl-accepting chemotaxis protein |
|  | L5014_16590 | chemotaxis protein (*CheA*) |
|  | L5014_16600 | methyl-accepting chemotaxis protein |
|  | L5014_17750 | methyl-accepting chemotaxis protein |
|  | L5014_19865 | methyl-accepting chemotaxis protein |
|  | L5014_20095 | methyl-accepting chemotaxis protein |
|  | L5014_22945 | methyl-accepting chemotaxis protein |
|  | L5014_23095 | methyl-accepting chemotaxis protein |
|  | L5014_25290 | methyl-accepting chemotaxis protein |
|  | L5014_27135 | methyl-accepting chemotaxis protein |
|  | L5014_27355 | methyl-accepting chemotaxis protein |
|  | L5014_30130 | methyl-accepting chemotaxis protein |
|  | L5014_35830 | methyl-accepting chemotaxis protein |
|  | L5014_16625 | chemotaxis response regulator (***CheY***) |
|  | L5014_01395 | chemotaxis response regulator protein-glutamate methylesterase |
|  | L5014_16620 | chemotaxis response regulator protein-glutamate methylesterase |
|  | L5014_13460 | type IV pili methyl-accepting chemotaxis transducer N-terminal domain-containing protein |
| Motility | L5014_04865 | flagellar protein **(*FliT*)** |
|  | L5014_07885 | flagellar brake protein |
|  | L5014_16675 | flagellar protein **(*FlgN*)** |
|  | L5014_16735 | flagellar brake protein |
|  | L5014_16835 | flagellar protein **(*FliT*)** |
|  | L5014_16690 | flagellar basal body protein |
|  | L5014_28770 | flagellar transcriptional regulator **(*FlhC*)** |
|  | L5014_08420 | flagellar transcriptional regulator **(*FlhC*)** |
|  | L5014_08425 | flagellar transcriptional regulator **(*FlhD*)** |
|  | L5014_12935 | flagellar transcriptional regulator **(*FlhD*)** |
|  | L5014_12940 | flagellar transcriptional regulator **(*FlhC*)** |
|  | L5014_16570 | flagellar transcriptional regulator **(*FlhD*)** |
|  | L5014_16580 | flagellar motor protein **(*MotB*)** |
|  | L5014_16650 | flagellar biosynthesis protein **(*FlhA*)** |
|  | L5014_16660 | flagellar biosynthesis protein **(*FlhF*)** |
|  | L5014_16700 | flagellar hook assembly protein **(*FlgD*)** |
|  | L5014_16705 | flagellar hook protein **(*FlgE*)** |
|  | L5014_16760 | flagellar biosynthetic protein **(*FliR*)** |
|  | L5014_16765 | flagellar biosynthesis protein **(*FliQ*)** |
|  | L5014_16775 | flagellar biosynthetic protein **(*FliO*)** |
|  | L5014_16800 | flagellar export protein **(*FliJ*)** |
|  | L5014_16810 | flagellar assembly protein **(*FliH*)** |
|  | L5014_16830 | flagellar export chaperone **(*FliS*)** |
|  | L5014_04870 | flagellar filament capping protein **(*FliD*)** |
|  | L5014_16575 | flagellar motor stator protein **(*MotA*)** |
|  | L5014_16730 | flagellar assembly peptidoglycan hydrolase **(*FlgJ*)** |
|  | L5014_16740 | flagellar hook-associated protein **(*FlgK*)** |
|  | L5014_16745 | flagellar hook-associated protein **(*FlgL*)** |
|  | L5014_16780 | flagellar motor switch protein **(*FliN*)** |
|  | L5014_16785 | flagellar motor switch protein **(*FliM*)** |
|  | L5014_16795 | flagellar hook-length control protein **(*FliK*)** |
|  | L5014_16805 | flagellar protein export ATPase **(*FliI*)** |
|  | L5014_16815 | flagellar motor switch protein **(*FliG*)** |
|  | L5014_16820 | flagellar M-ring protein **(*FliF*)** |
|  | L5014_16840 | flagellar hook-length control protein **(*FliK*)** |
|  | L5014_16680 | flagellar biosynthesis anti-sigma factor **(*FlgM*)** |
|  | L5014_16695 | flagellar basal body rod protein **(*FlgC*)** |
|  | L5014_16710 | flagellar basal-body rod protein **(*FlgF*)** |
|  | L5014_16715 | flagellar basal-body rod protein **(*FlgG*)** |
|  | L5014_16725 | flagellar basal body P-ring protein **(*FlgI*)** |
|  | L5014_16790 | flagellar basal body-associated protein **(*FliL*)** |
|  | L5014_16720 | flagellar basal body L-ring protein **(*FlgH*)** |
|  | L5014_16825 | flagellar hook-basal body complex protein **(*FliE*)** |
|  | L5014_16685 | flagellar basal body P-ring formation protein **(*FlgA*)** |
|  | L5014_16770 | flagellar type III secretion system pore protein **(*FliP*)** |

**Table S4.** Putative genes associated quorum sensing in the genome of strain RG36^T^.

| **Function** | **Locus tag** | **Annotation** |
| --- | --- | --- |
| Quorum sensing | L5014_04715 | quorum-sensing autoinducer CAI-1 synthase **(*CqsA*)** |
|  | L5014_30675 | autoinducer 2 ABC transporter substrate-binding protein |
|  | L5014_08505 | transcriptional regulator **(*LuxR*)** |
|  | L5014_23625 | transcriptional regulator **(*LuxR*)** |
|  | L5014_00360 | C-terminal-related transcriptional regulator **(*LuxR*)** |
|  | L5014_22075 | C-terminal-related transcriptional regulator **(*LuxR*)** |
|  | L5014_25595 | C-terminal-related transcriptional regulator **(*LuxR*)** |
|  | L5014_29430 | C-terminal-related transcriptional regulator **(*LuxR*)** |
|  | L5014_37440 | C-terminal-related transcriptional regulator **(*LuxR*)** |
|  | L5014_37470 | C-terminal-related transcriptional regulator **(*LuxR*)** |

**Table S5.** Putative genes associated with secretion systems in the genome of strain RG36^T^.

| **Type** | **Locus tag** | **Protein Name** |
| --- | --- | --- |
| I | L5014_04220 | HlyD family secretion protein |
|  | L5014_04625 | HlyD family secretion protein |
|  | L5014_07730 | HlyD family secretion protein |
|  | L5014_18520 | HlyD family secretion protein |
|  | L5014_27260 | HlyD family secretion protein |
|  | L5014_30830 | HlyD family secretion protein |
|  | L5014_19925 | type I secretion system permease/ATPase |
|  | L5014_30435 | HlyD family type I secretion periplasmic adaptor subunit |
|  | L5014_30440 | type I secretion system permease/ATPase |
| II and III | L5014_27920 | type II secretion system protein |
|  | L5014_31355 | type II secretion system protein M |
|  | L5014_31360 | type II secretion system protein N |
|  | L5014_11265 | type II secretion system F family protein |
|  | L5014_11270 | type II secretion system F family protein |
|  | L5014_18135 | type II secretion system F family protein |
|  | L5014_18140 | type II secretion system F family protein |
|  | L5014_18185 | type II secretion system F family protein |
|  | L5014_18190 | type II secretion system F family protein |
|  | L5014_31310 | type II secretion system ATPase **(*GspE*)** |
|  | L5014_31350 | type II secretion system protein **(*GspL*)** |
|  | L5014_33690 | type II secretion system F family protein |
|  | L5014_00410 | type II secretion system major pseudopilin **(*GspG*)** |
|  | L5014_31325 | type II secretion system major pseudopilin **(*GspG*)** |
|  | L5014_31335 | type II secretion system minor pseudopilin **(*GspI*)** |
|  | L5014_31345 | type II secretion system minor pseudopilin **(*GspK*)** |
|  | L5014_31320 | general secretion pathway protein **(*GspC*)** |
|  | L5014_31305 | type II secretion system secretin **(*GspD*)** |
|  | L5014_18115 | type II and III secretion system protein family protein |
|  | L5014_18210 | type II and III secretion system protein family protein |
|  | L5014_31315 | type II secretion system inner membrane protein **(*GspF*)** |
| III | L5014_16845 | EscU/YscU/HrcU family type III secretion system export apparatus switch protein |
|  | L5014_16770 | flagellar type III secretion system pore protein **(*FliP*)** |
| IV | L5014_23545 | type IV secretion system protein |
|  | L5014_23555 | type IV secretion system protein |
|  | L5014_27865 | type IV secretion system protein |
|  | L5014_28525 | type IV secretion system protein |
|  | L5014_28535 | type IV secretion system protein **(*VirB4*)** |
|  | L5014_31745 | DotU family type IV secretion system protein |
|  | L5014_23535 | type IV secretion system protein **(*VirB10*)** |
|  | L5014_23585 | type IV secretion system protein **(*VirB*)** |
|  | L5014_28540 | type IV secretion system protein **(*VirB*)** |
| VI | L5014_00795 | type VI secretion protein **(*VasK*)** |
|  | L5014_21760 | type VI secretion protein **(*VasK*)** |
|  | L5014_22720 | type VI secretion protein **(*VasK*)** |
|  | L5014_04495 | type VI secretion system protein **(*DotU*)** |
|  | L5014_04520 | type VI secretion system ATPase **(*TssH*)** |
|  | L5014_04545 | type VI secretion system tube protein **(*Hcp*)** |
|  | L5014_04560 | type VI secretion system protein **(*TssA*)** |
|  | L5014_21695 | type VI secretion system ATPase **(*TssH*)** |
|  | L5014_21700 | type VI secretion system effector **(*Hcp*)** |
|  | L5014_21765 | type VI secretion system protein **(*TssA*)** |
|  | L5014_21780 | type VI secretion system lipoprotein **(*TssJ*)** |
|  | L5014_22705 | type VI secretion system lipoprotein **(*TssJ*)** |
|  | L5014_25955 | type VI secretion system lipoprotein **(*TssJ*)** |
|  | L5014_25965 | type VI secretion system protein **(*DotU*)** |
|  | L5014_25985 | type VI secretion system protein **(*TssA*)** |
|  | L5014_26000 | type VI secretion system tube protein **(*Hcp*)** |
|  | L5014_27360 | type VI secretion system protein **(*TssA*)** |
|  | L5014_27375 | type VI secretion system tube protein **(*Hcp*)** |
|  | L5014_27425 | type VI secretion system lipoprotein **(*TssJ*)** |
|  | L5014_00755 | type VI secretion system tip protein **(*VgrG*)** |
|  | L5014_00870 | type VI secretion system tube protein **(*Hcp*)** |
|  | L5014_04485 | type VI secretion system-associated protein **(*TagF*)** |
|  | L5014_04490 | type VI secretion system membrane subunit **(*TssM*)** |
|  | L5014_04500 | type VI secretion system baseplate subunit **(*TssK*)** |
|  | L5014_04510 | type VI secretion system tip protein **(*VgrG*)** |
|  | L5014_04525 | type VI secretion system baseplate subunit **(*TssG*)** |
|  | L5014_04530 | type VI secretion system baseplate subunit **(*TssF*)** |
|  | L5014_04535 | type VI secretion system baseplate subunit **(*TssE*)** |
|  | L5014_17765 | type VI secretion system tube protein **(*Hcp*)** |
|  | L5014_19880 | type VI secretion system tube protein **(*Hcp*)** |
|  | L5014_19920 | family type I secretion periplasmic adaptor subunit **(*HlyD*)** |
|  | L5014_20220 | type VI secretion system tip protein **(*VgrG*)** |
|  | L5014_21690 | type VI secretion system baseplate subunit **(*TssE*)** |
|  | L5014_21710 | family type IV/VI secretion system protein **(*DotU*)** |
|  | L5014_21715 | type VI secretion system baseplate subunit **(*TssK*)** |
|  | L5014_21730 | type VI secretion system tip protein **(*VgrG*)** |
|  | L5014_21770 | type VI secretion system baseplate subunit **(*TssF*)** |
|  | L5014_21775 | type VI secretion system baseplate subunit **(*TssG*)** |
|  | L5014_22710 | type VI secretion system baseplate subunit **(*TssG*)** |
|  | L5014_22715 | type VI secretion system baseplate subunit **(*TssF*)** |
|  | L5014_25970 | type VI secretion system membrane subunit **(*TssM*)** |
|  | L5014_25975 | type VI secretion system-associated protein **(*TagF*)** |
|  | L5014_26005 | type VI secretion system baseplate subunit **(*TssE*)** |
|  | L5014_26010 | type VI secretion system baseplate subunit **(*TssF*)** |
|  | L5014_26015 | type VI secretion system baseplate subunit **(*TssG*)** |
|  | L5014_26020 | type VI secretion system tip protein **(*VgrG*)** |
|  | L5014_27380 | type VI secretion system baseplate subunit **(*TssE*)** |
|  | L5014_27385 | type VI secretion system baseplate subunit **(*TssF*)** |
|  | L5014_27390 | type VI secretion system baseplate subunit **(*TssG*)** |
|  | L5014_27395 | type VI secretion system tip protein **(*VgrG*)** |
|  | L5014_27400 | type VI secretion system tip protein **(*VgrG*)** |
|  | L5014_27430 | type VI secretion system baseplate subunit **(*TssK*)** |
|  | L5014_27440 | type VI secretion system membrane subunit **(*TssM*)** |
|  | L5014_30030 | type VI secretion system tip protein **(*VgrG*)** |
|  | L5014_31740 | type VI secretion system baseplate subunit **(*TssE*)** |
|  | L5014_31745 | family type IV/VI secretion system protein **(*DotU*)** |
|  | L5014_31750 | type VI secretion system baseplate subunit **(*TssK*)** |
|  | L5014_32780 | type VI secretion system tube protein **(*Hcp*)** |
|  | L5014_36195 | type VI secretion system tip protein, partial **(*VgrG*)** |
|  | L5014_38240 | type VI secretion system tip protein **(*VgrG*)** |
|  | L5014_38430 | type VI secretion system tip protein, partial **(*VgrG*)** |
|  | L5014_04550 | type VI secretion system contractile sheath large subunit **(*TssC*)** |
|  | L5014_04555 | type VI secretion system contractile sheath small subunit **(*TssB*)** |
|  | L5014_21720 | type VI secretion system contractile sheath small subunit **(*TssB*)** |
|  | L5014_21725 | type VI secretion system contractile sheath large subunit **(*TssC*)** |
|  | L5014_25990 | type VI secretion system contractile sheath small subunit **(*TssB*)** |
|  | L5014_25995 | type VI secretion system contractile sheath large subunit **(*TssC*)** |
|  | L5014_27365 | type VI secretion system contractile sheath small subunit **(*TssB*)** |
|  | L5014_27370 | type VI secretion system contractile sheath large subunit **(*TssC*)** |
|  | L5014_31755 | type VI secretion system contractile sheath large subunit **(*TssC*)** |
|  | L5014_04505 | type VI secretion system-associated FHA domain protein **(*TagH*)** |
|  | L5014_25945 | type VI secretion system-associated FHA domain protein **(*TagH*)** |

##
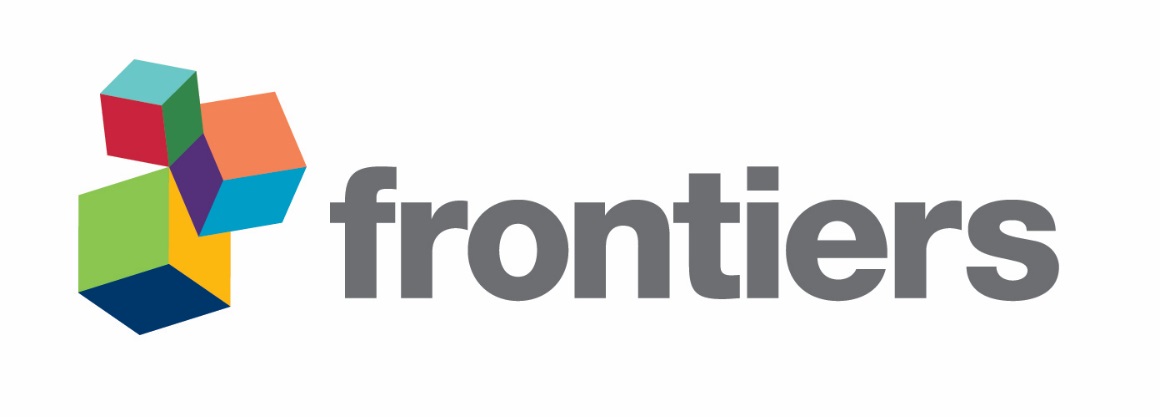

Supplement: Supplementary file 1 [file Data_Sheet_1.docx]
